# Gut *Subdoligranulum variabile* ameliorates rheumatoid arthritis by promoting TSG-6 synthesis from joint cells

**DOI:** 10.3389/fimmu.2024.1418717

**Published:** 2024-06-10

**Authors:** Hongfeng Li, Junhui Dai, Changying Zhao, Tianqi Hu, Guoping Zhao, Qinghua Wang, Lei Zhang

**Affiliations:** ^1^ Microbiome-X, School of Public Health, Cheeloo College of Medicine, Shandong University, Jinan, China; ^2^ Department of Health Inspection and Quarantine, School of Public Health, Cheeloo College of Medicine, Shandong University, Jinan, Shandong, China; ^3^ State Key Laboratory of Microbial Technology, Shandong University, Qingdao, China; ^4^ Chinese Academy of Sciences Key Laboratory of Computational Biology, Bio-Med Big Data Center, Shanghai Institute of Nutrition and Health, University of Chinese Academy of Sciences, Chinese Academy of Sciences, Shanghai, China; ^5^ School of Biological Science and Technology, University of Jinan, Jinan, China

**Keywords:** rheumatoid arthritis, gut microbiota, mendelian randomization, pathway discovery, tumor necrosis factor-inducible gene 6 protein

## Abstract

**Background:**

A burgeoning body of evidence has substantiated the association between alterations in the composition of the gut microbiota and rheumatoid arthritis (RA). Nevertheless, our understanding of the intricate mechanisms underpinning this association is limited.

**Methods:**

To investigate whether the gut microbiota influences the pathogenesis of RA through metabolism or immunity, we performed rigorous synthesis analyses using aggregated statistics from published genome-wide association studies (GWAS) using two-sample Mendelian randomization (MR) and mediated MR techniques, including two-step MR and multivariate MR analyses. Subsequently, we conducted *in vitro* cellular validation of the analyzed Microbial-Cytokine-RA pathway. We determined the optimal culture conditions through co-culture experiments involving concentration and time. Cell Counting Kit-8 (CCK-8) assays were employed to assess cellular viability, and enzyme-linked immunosorbent assays (ELISA) were performed to assess tumor necrosis factor-inducible gene 6 protein (TSG-6) and tumor necrosis factor-α (TNF-α) levels.

**Results:**

Our univariable MR results confirmed 15 microbial traits, 7 metabolites and 2 cytokines that may be causally associated with RA (*P*_FDR_ < 0.05). Mediation analysis revealed that microbial traits influence the risk of RA through metabolite or cytokine (proportion mediated: 7.75% - 58.22%). *In vitro* experiments demonstrated that TSG-6 was highly expressed in the *Subdoligranulum variabile* treatment group and was correlated with decreased RA severity (reduced TNF-α expression). Silencing the TSG-6 gene significantly increased TNF-α expression, regardless of treatment with *S. variabile*. Additionally, *S. variabile*-secreted exosomes exhibited the same effect.

**Conclusion:**

The results of this study suggest that *S. variabile* has the potential to promote TSG-6 secretion, thereby reducing RA inflammation.

## 1 Introduction

Rheumatoid arthritis (RA) is a chronic autoimmune disease characterized by progressive articular damage and functional loss. It can also lead to vascular, metabolic, bone, and psychological comorbidities (1). The development of RA is believed to be influenced by complex interactions between genetic and environmental factors. However, the exact mechanisms underlying the pathogenesis of RA remain unclear, making it difficult to detect the disease at an early stage and differentiate it from other autoimmune diseases (2). Given the current lack of compelling specificity and sensitivity in RA diagnostic methods, elucidating the underlying mechanisms is critical for facilitating timely diagnosis and intervention strategies (3).

In recent years, there has been increasing interest in exploring the relationship between the gut microbiota composition and the risk of autoimmune diseases (4). Emerging sequencing-based methodologies have revealed notable differences in the gut microbiota composition between patients diagnosed with autoimmune diseases such as RA (5), multiple sclerosis (6), type 1 diabetes (7), and systemic lupus erythematosus (8), and their healthy counterparts. Interactions between the microbiome and the immune system have been observed to exert influence on the progression of immune diseases (9). Various mechanisms have been identified through which the microbiota may contribute to these diseases, including molecular mimicry, direct interactions with immune cells, and alterations in the abundance of immunomodulatory microbial metabolites (10). Specifically, certain groups of metabolites, such as short-chain fatty acids (SCFAs) and tryptophan metabolites, have been extensively studied and found to have significant impacts on immune system physiology (11). These metabolites exhibit specific effects at both the cellular and systemic levels by interacting with various receptors present in immune cells. An imbalance in the gut microbiota affects the levels of metabolites (12) and subsequently then leads to the occurrence of autoimmune diseases (13). A multitude of studies have unequivocally established a robust correlation between the pathogenesis of RA and gut microbiota-derived metabolites, including but not limited to SCFAs, bile acids, and tryptophan and its derivatives (14, 15). Furthermore, it has been shown that gut microbes can reduce inflammation by increasing the levels of anti-inflammatory factors in the body, thereby mitigating inflammation (16). A series of epidemiological, clinical and animal experiments have indicated a relationship between the gut microbiota and the pathogenesis of RA (13, 17). Specifically, several researchers have indicated that certain specific gut symbiotic bacteria can alleviate RA (18, 19). However, a substantial portion of correlation studies have failed to elucidate the intricate causal mechanisms underlying the Gut-Joint axis.

In this context, Mendelian randomization (MR) is an excellent approach for probing the causal association between the gut microbiota and RA. Most Mendelian randomization (MR) studies assess causality by using genetic variation as instrumental variables (IVs), which overcome the bias due to confounding and reverse causation (20). In the mediation section, two-step MR (21) and multivariable MR (MVMR) (22) are two different MR approaches used to decompose indirect and direct effects.

In this study, we first conducted a two-sample MR analysis using aggregated summary statistics from other studies. Additionally, we employed two-step MR and MVMR analyses to explore potential causal pathways through which gut microbial profiles may influence RA, either via metabolites or cytokines. Finally, we selected the Microbial-Cytokine-RA pathway and conducted preliminary validation using *in vitro* cell experiments.

## 2 Methods

### 2.1 Data sources

The MR research used publicly accessible datasets containing summary statistics from genome-wide association studies (GWAS) (**Table 1**; **Supplementary Table 1**). Details regarding the quality control (QC), imputation, and GWAS procedures for each study have been previously described elsewhere (23–29). The GWAS summary data for RA were obtained from the UK Biobank, and consisted of 1523 cases and 461487 controls (23). The genetic data for gut microbial taxa were obtained from previously published GWAS analyses, which included five independent cohorts from German biobanks located in different regions of Germany, with a total sample size of 8956 and 430 univariate microbial features included in the GWAS (24) (**Table 1**; **Supplementary Table 1**). The genome-wide summary statistics for blood metabolites were obtained from the study conducted by Shin et al. (25), which included 275 metabolites profiled in plasma or serum from 7824 adult individuals in two European population studies (**Table 1**; **Supplementary Table 1**). The GWAS summary statistics for each cytokine were extracted from publicly available GWAS analyses (26–28).

**Table 1:** GWAS samples used in this study.

Phenotype	Trait size	Sample size	PMID	Reference
Rheumatoid Arthritis	1	463010 (1523 cases, 461487 controls)	34662886	Backman, Joshua D et al. (23)
Gut microbial traits	430	8956	33462482	Ruhlemann et al. (24)
Blood metabolites	275	7824	24816252	Shin et al. (25)
Cytokines	52	8189 3301 3394	32641083 29875488 28369058	Hillary et al. (26); Sun et al. (27); Folkersen et al. (28)

GWAS, genome-wide association study; PMID, Pubmed ID.

### 2.2 Selection of instrumental variables

The development of a robust IV that fulfills the following criteria (1): the IV demonstrates an association with the exposure under investigation, (2) the IV is independent of any confounders in the association between the exposure and the outcome, and (3) the IV affects only the outcome via the exposure pathway. Therefore, we used a genome-wide significance threshold (*P* < 5 × 10^-8^) to select metabolic, cytokine, and RA-associated single nucleotide polymorphisms (SNPs). Previous studies have shown that SNPs associated with *P* < 1×10^-5^ had the greatest variance in microbial traits (24). To ensure the reliability and accuracy of our conclusions, we employed two thresholds (*P* < 5×10^-8^ and *P* < 1×10^-5^) to select IVs for microbial traits. Linkage disequilibrium among SNPs was subsequently estimated using the clump parameter in PLINK (1.9) software to select independent genetic variants (window size = 10,000 kb, *R* < 0.01) and to exclude palindromic SNP with non-derivative allele frequency (MAF > 0.3).

The *F*-statistic was calculated to assess the effectiveness of the IVs. IVs with *F*-statistics less than 10 were deemed weak and subsequently excluded. Furthermore, we conducted a search in the PhenoScanner GWAS database (version 2; http://phenoscanner.medschl.cam.ac.uk) to identify any potential instances of pleiotropy between exposure-associated SNPs and associated traits (30).

### 2.3 Mendelian randomization analyses

To assess the plausible causal relationships among the gut microbiota, metabolites, cytokines, and RA, we conducted two-sample MR analyses (31). Several MR methods have been used, including the Wald ratio (32), maximum likelihood, inverse-variance weighted (multiplicative random effects) (33), and weighted median methods. The IVW or Wald ratio was used as the primary outcome measure.

To validate the robustness of our findings, we utilized the MR Steiger test as a methodological tool to ascertain the probable orientation of causality between the exposures and outcomes. For microbiota, metabolites, and cytokines showing evidence of a causal effect on RA, we used the “coloc” method to investigate whether the same genetic variant influencing these factors also influenced RA (34). We set a threshold of PP.H4.abf > 0.8 to filter comparisons with strong support for an association with both traits. Additionally, we conducted a series of sensitivity analyses to address violations of the MR assumptions. The Cochran Q statistic was employed to evaluate the collective heterogeneity exhibited by the chosen SNPs. MR−Egger regression and leave-one-out analyses were employed to detect and account for potential horizontal pleiotropy (35). Furthermore, we employed MR-PRESSO (Mendelian Randomization Pleiotropy Residual Sum and Outliers) (36) to identify and correct for potential outliers (*P* < 0.05). Outliers were removed in an attempt to re-estimate the original exposure−outcome relationship and minimize bias in the MR estimates (31). Finally, we performed a reverse MR analysis to evaluate whether there was genetic evidence for a reverse causal effect, where RA subsequently altered the microbiota, metabolites, and cytokines. We conducted and documented the MR analysis in strict compliance with the STROBE-MR guidelines (20) and included a comprehensive list of the STROBE-MR guidelines in the **Supplementary File**.

To investigate the potential biological significance of the gut microbiota in the pathogenesis of RA, we conducted Kyoto Encyclopedia of Genes and Genomes (KEGG) enrichment analysis based on the lead SNPs associated with all identified microbiota. We mapped the lead SNPs of the causative microbiota identified in various RA phenotypes to neighboring genes. Statistical significance was determined using the Benjamini−Hochberg method, with analyses yielding a *p*-value < 0.05 considered as statistically significant.

### 2.4 Mediation analysis

For blood metabolites and cytokines for which there is evidence of a causal effect on RA and which are influenced by the gut microbiota, we used a two-step MR (21) and MVMR (22) to investigate mediation.

**Figure 1:**
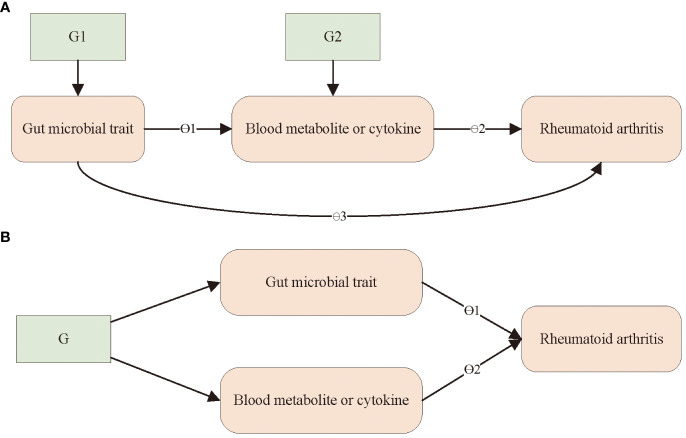
Outline of the steps of the mediation analysis. **(A)** A two-step Mendelian randomization analysis was used to assess the mediating role of blood metabolites or immune factors in the effect of gut microbiota on RA. θ1 = step 1; θ2 = step 2; indirect effect = θ1× θ2 (product of coefficients); direct effect = θ3; total causal effect = θ3 + θ1 × θ2; G1 and G2, instrumental variable. **(B)** A multivariable Mendelian randomization analysis was used to assess the mediating role of blood metabolites and immune factors in the effect of gut microbiota on RA. direct effect of gut microbial trait = θ1; direct effect of metabolite or immune factor = θ2. G, instrumental variable.

For blood metabolites where there was evidence of microbial traits influencing metabolites, which in turn influenced RA, we employed the product-of-coefficients method (37) to assess the indirect impact of the microbiota on RA through blood metabolites. The standard errors for the indirect effect were computed using the delta method (38). An alternative approach in MR analysis, known as MVMR, can be applied to assess mediation. This method enables researchers to ascertain the direct effect of an exposure on an outcome, which can be subtracted from the total effect to obtain an estimate of the indirect effect (difference-in-coefficients method) (37). We used MVMR to evaluate the direct effects of gut microbial traits and the identified blood metabolites on RA by including genetic proxies for microbial traits and each metabolite in multivariable models. This approach is outlined in **Figure 1**. These analyses were conducted employing the mv_multiple function in TwoSampleMR, the R package in the MR-Base platform. Conditional *F*-statistics were calculated for each exposed phenotype to assess the combined power of the instruments in MVMR analyses. Finally, we estimated the proportion mediated by dividing the mediated effect by the total effect, as previously described. The aforementioned method was also applied to cytokines.

### 2.5 Cell and bacterial cultures

In exploratory experiments, we attempted to validate the MR analysis results (Microbial-Cytokine-RA) at the cellular level. We selected cells that are capable of producing TSG-6 within the intra-articular environment, encompassing chondrocytes and synoviocytes (39). Moreover, fibroblasts emerged as a significant contributor to synovial inflammation and joint damage (40). Immortalized human chondrocytes (HC) were purchased from Hefei Wanwu Biotechnology Co., Ltd (Hefei, China). Human rheumatoid arthritis fibroblast-like synoviocytes (HFLS-RA) were purchased from Shanghai Honsun Biological Technology Co., Ltd (Shanghai, China). These two types of cells were cultured in Dulbecco’ s Modified Eagle Medium (DMEM, Sigma, USA) supplemented 10% Fetal Bovine Serum (FBS, Gibco, USA) and 1% Penicillin-Streptomycin Solution (PS, Gibco, USA) at 37°C; in a humidified 5% CO_2_ atmosphere, and the medium was routinely replaced every 2–3 days. For all experiments in this study, cells were used within 7 passages.

*Subdoligranulum variabile* DSM 15176^T^ (*S. variabile*) was obtained from the Leibniz Institute DSMZ-German Collection of Microorganisms and Cell Cultures GmbH (Braunschweig, Germany). The strain was grown anaerobically (N_2_/CO_2_ 80:20 v/v) in Chopped Meat Carbohydrate Broth (CMC, Topu, China) supplemented with Hematin chloride solution, vitamin K_3_ solution, and Chopped Meat. As in previous studies, *S. variabile* was not enumerated on solid medium (41). Therefore, the enumeration of live and cultivable bacteria was conducted using the most likely number calculation associated with the dilution to extinction method. Liquid cultures were diluted in cell culture medium to the required concentration before each experiment.

### 2.6 Co-culture of *S. variabile* and HC or HFLS-RA

HC or HFLS-RA cells were seeded at a density of 7×10^4^ cells/well in 24-well plates for 12 hours. Subsequently, when the cells reached 80–90% confluence, *S. variabile* (4×10^2^-4×10^4^ CFU/mL) (42) or exosomes (1–13 µg/mL) were added to the medium. To determine the optimal culture concentration and time, the levels of tumor necrosis factor-inducible gene 6 protein (TSG-6) and tumor necrosis factor-α (TNF-α) in the cell supernatant were measured after co-culture for 6–24 hours (**Supplementary Figure 2**).

### 2.7 Cell cytotoxicity assay

The cytotoxicity of *S. variabile* was assessed utilizing a Cell Counting Kit-8 (CCK-8; Vazyme, China) following the manufacturer’s guidelines. Briefly, HC or HFLS-RA (1×10^4^ cells per well) were seeded into 96-well plates and incubated at 37°C and 5% CO_2_ for 12 hours. Next, *S. variabile*-containing cell culture medium (100 µL) was added to the cultured cells. After an 8-hour incubation period, 10 µL of CCK-8 solution was added to each the well, followed by an additional 2-hour incubation at 37°C. The control group comprised both cells and CCK8, whereas the blank group consisted solely of CCK8. Subsequently, the absorbance was measured at 450 nm using an absorbance microplate reader (Bio Tek). The viability of cells (%) was calculated by the following formula: viability (%) = (mean OD of cells of the experimental group - mean OD of blank)/(mean OD of cells of the control group - mean OD of blank) × 100.

### 2.8 Exosome isolation using EXODUS

To obtain exosomes, *S. variabile* were incubated in liquid medium for 12 hours. Subsequently, the bacterial solution was initially centrifuged at 2,000×g for 10 minutes at 4°C. The resulting pellet was discarded, and the resulting supernatant was centrifuged again at 12,000×g for 20 minutes at 4°C. After that, the supernatant was filtered through 0.22 μm filters, and the filtrate was collected. The prepared sample tube was loaded in the sample holder, and the EXODUS system was started (43). Using a pipette, the exosome solution was collected from the EXODUS device and reconstituted to a volume of 600 µL in a 1.5 mL centrifugation tube with PBS after completing the sample analysis. The protein content of exosome was measured by bicinchoninic acid kit (BCA; ThermoFisher, USA) according to the manufacturer’s instructions. The isolated exosomes were immediately stored at -80°C until further experiments.

### 2.9 Small interfering RNA transfection

To generate small interfering RNA (siRNA) against TSG-6, GenePharma (Shanghai, China) chemically synthesized and annealed the siRNA sequences to form the siRNA duplex. The sense sequence was 5’-GGCGGUGUGUGAAUUUGAATT-3’; while the antisense sequence was 5’-UUCAAAUUCACACACCGCCTT-3’. Prior to siRNA treatment with Lipofectamine™ 3000 siRNA transfection reagent (ThermoFisher, USA) and siRNA against TSG-6, 1×10^5^ cells/well HC or HFLS-RA were seeded and cultured for 24 hours in 6-well plates.

### 2.10 Transwell co-culture models

The co-culture model of *S. variabile* and human cell lines was established as follows. HC were seeded at a density of 1.6×10^5^ cells/well into the lower chamber, and HFLS-RA were added at a density of 4×10^4^ cells/well to the upper chamber of the transwell (0.4 μm pores, Corning, Germany). *S. variabile* (6×10^2^ CFU/mL) was then added to the medium in the lower chamber when the cells reached 80–90% confluence. After incubating the cultures for two hours, the upper chamber was reinserted into the transwell system to create a co-culture model, and subsequent analyses were conducted after 8 hours of co-culture.

### 2.11 Measurement of secreted TSG-6 and TNF-α

After co-culturing bacteria with human cell lines, the levels of TSG-6 and TNF-α in the cell supernatants were quantified by ELISA (Solarbio, China). Briefly, the supernatant from the co-culture is transferred to a centrifuge tube and centrifuged at 2,500 rpm for 25 min at 4°C. Cytokine concentrations were determined using the standard curve method and following the manufacturer’s instructions. Absorbance was measured at 450 nm. The rang limits of detection for these assays are 0.625‐20 ng/mL, 2.5–80 pg/mL for TSG-6 and TNF-α, respectively.

### 2.12 Statistics

**Figure 2:**
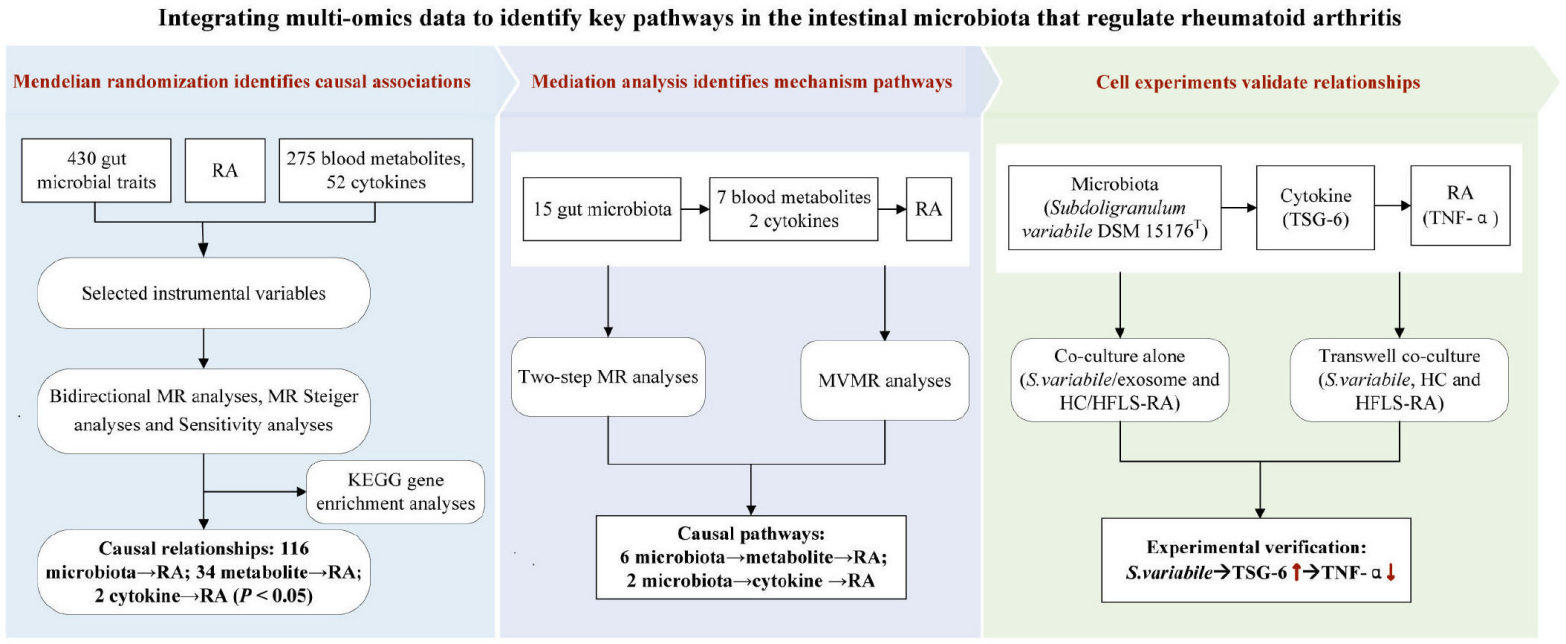
The whole workflow of analysis. RA, rheumatoid arthritis; MR, Mendelian randomization; GWAS, genome-wide association study; SNPs, single nucleotide polymorphisms; MR-PRESSO, Mendelian Randomization Multiiotropy Residual Sum and Outlier; MVMR, multivariable MR.

The entire workflow of the MR analysis is depicted in **Figure 2**. All analyses were conducted using R, version 4.0.3 (http://www.r-project.org). The “MendelianRandomization” package (version 0.4.3) was utilized for MR analysis and MVMR analysis. MR-PRESSO was carried out using the “MR-PRESSO” package. KEGG enrichment analysis was performed using the online tool “Sangerbox 3.0” (http://vip.sangerbox.com/). Experimental data analysis was conducted utilizing GraphPad Prism 10 software (San Diego, CA, USA). Normally distributed experimental results, as determined by the Shapiro−Wilk normality test, were analyzed using one-way ANOVA with Bonferroni *post hoc* correction for more than 2 groups.

## 3 Results

First, 23 SNPs associated with the microbiota were identified at a significance level of *P* < 5×10^-8^. When utilizing a loose threshold of 1×10^-5^ to select IVs, a total of 4332 SNPs were found to be associated with 429 microbiota. The *F*-statistics of these IVs ranged from 19.526 to 1498.702, all of which were above the threshold of 10, indicating no evidence of weak instrument bias. This demonstrates the robustness and reliability of the IVs used in the study.

### 3.1 Gut microbial traits with RA risk

**Figure 3:**
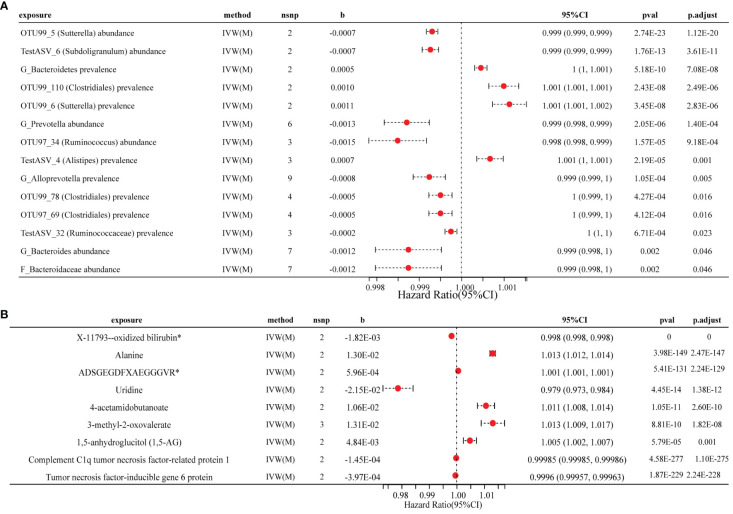
Forest plot of the causal effects of three factors on RA. **(A)** Causal effect of gut microbial traits on RA forest plot. **(B)** Forest plot of the causal association between metabolites, cytokines and RA. RA, rheumatoid arthritis; IVW(M), Inverse variance weighted (multiplicative random effects); nsnp, the number of IVs; 95% CI, OR (95% confidence interval); pval of the intercept from the method; p.adjust, pval by the Benjamini-Hochberg method.

In the set of IVs (*P* < 1×10^−5^), we identified 15 microbota that mainly belonged to the orders *Burkholderiales*, *Clostridiales*, and *Bacteroidales*, which were causally associated with RA (**Figure 3A**; **Supplementary Table 2**). Among these microbial traits, 11 were found to be protective factors for RA (*P_BH_* < 0.05). Specifically, we observed that the OTU99_5 (*Sutterella*) abundance (odds ratio (OR) = 0.999, 95% CI: 0.999–0.999, *P_BH_* = 1.12×10^−20^), TestASV_6 (*Subdoligranulum*) abundance (OR = 0.999, 95% CI: 0.999–0.999, *P_BH_* = 3.61×10^−11^), OTU99_78 (*Clostridiales*) prevalence (OR = 0.999, 95% CI: 0.999–1.000, *P_BH_* = 0.016) and TestASV_32 (*Ruminococcaceae*) prevalence (OR = 0.999, 95% CI: 0.9996–1.000, *P_BH_* = 0.023) were causally associated with RA. Additionally, we found that *G_Bacteroidetes* prevalence (OR = 1.0004, 95% CI: 1.000–1.001, *P_BH_* = 7.08×10^−8^) and TestASV_4 (*Alistipes*) prevalence (OR = 1.0007, 95% CI: 1.000–1.001, *P_BH_* = 0.001) were also observed to be causally associated to RA. These findings highlight the potential role of these specific microbiota in the development and progression of RA.

In the other group IV, only a small number of gut microbiota were found to be significantly associated with RA (*P* < 5×10^−8^), no definitive evidence of a causal relationship between microbiota and RA (**Supplementary Table 12**). When conducting reverse MR analysis of gut microbiota and RA, we identified RA that showed a causal association with 83 microbial traits (**Supplementary Table 3**).

Enrichment analysis of microbial taxa using KEGG revealed a significant enrichment of several key regulatory pathways. We identified 11 KEGG biological processes that were involved in RA, such as Axon guidance and ErbB signaling pathways (**Supplementary Figure 1**). This finding highlights the importance of these pathways in the pathogenesis of RA and contributes to the growing body of academic knowledge in this field.

### 3.2 Blood metabolites and cytokines with RA risk

The results of the MR analyses revealed that seven metabolites are significantly associated with RA risk (*P_BH_* < 0.05) (**Figure 3B**; **Supplementary Table 4**). We observed a positive causal effect of three metabolites, namely 3-methyl-2-oxovalerate (OR = 1.013, 95% CI: 1.010–1.017, *P_BH_* = 1.82×10^-8^), ADSGEGDFXAEGGGVR* (OR = 1.0006, 95% CI: 1.0005–1.0006, *P_BH_* = 2.24×10^-129^) and alanine (OR = 1.013, 95% CI: 1.012–1.014, *P_BH_* = 2.47×10^-147^) on RA. Furthermore, we identified a significant negative causal effect of X-11793–oxidized bilirubin* (OR = 0.998, 95% CI: 0.9981–0.9982, *P_BH_* < 0.001) on RA.

MR analyses revealed negative causal inferences for complement C1q tumor necrosis factor-related protein 1 (CTRP1) (OR = 0.9999, 95% CI: 0.99984–0.999986, *P_BH_* = 1.10×10^-275^) and tumor necrosis factor-inducible gene 6 protein (OR = 0.9996, 95% CI: 0.99958–0.99963, *P_BH_* = 2.24×10^-228^) with RA (**Figure 3B**; **Supplementary Table 4**). Additionally, we identified a causal relationship in the opposite direction for 10 cytokines and RA (**Supplementary Table 5**). Unfortunately, we were unable to assess reverse causality between metabolites and RA due to the absence of matching SNPs for exposure and outcome.

### 3.3 Sensitivity analysis

The Cochran’s Q and MR-PRESSO tests revealed a significant presence of horizontal pleiotropy between OTU99_110 (*Clostridiales*) abundance and RA (*P* = 0.047). No signs of heterogeneity or pleiotropy were observed among the genetic instrumental variables concerning the other outcomes (all *P* > 0.05, **Supplementary Table 6**). Since most metabolites and cytokines had fewer than two IVs, additional analyses were conducted to assess the robustness of the causal findings. MR Steiger analysis indicated a forward causal direction from exposure to outcome (all *P* < 2.09×10^-5^, **Supplementary Table 7**), and Colocation analysis demonstrated that the variation in exposure and outcome could not be attributed to the same underlying genetic variation (based on PP.H4.abf < 80%, **Supplementary Table 8**), suggesting that the IVW regression returns unbiased estimates for the causal effect.

### 3.4 Mediation analyses: the effect of Gut-Metabolism/Cytokine-Joint Axis on RA

**Figure 4:**
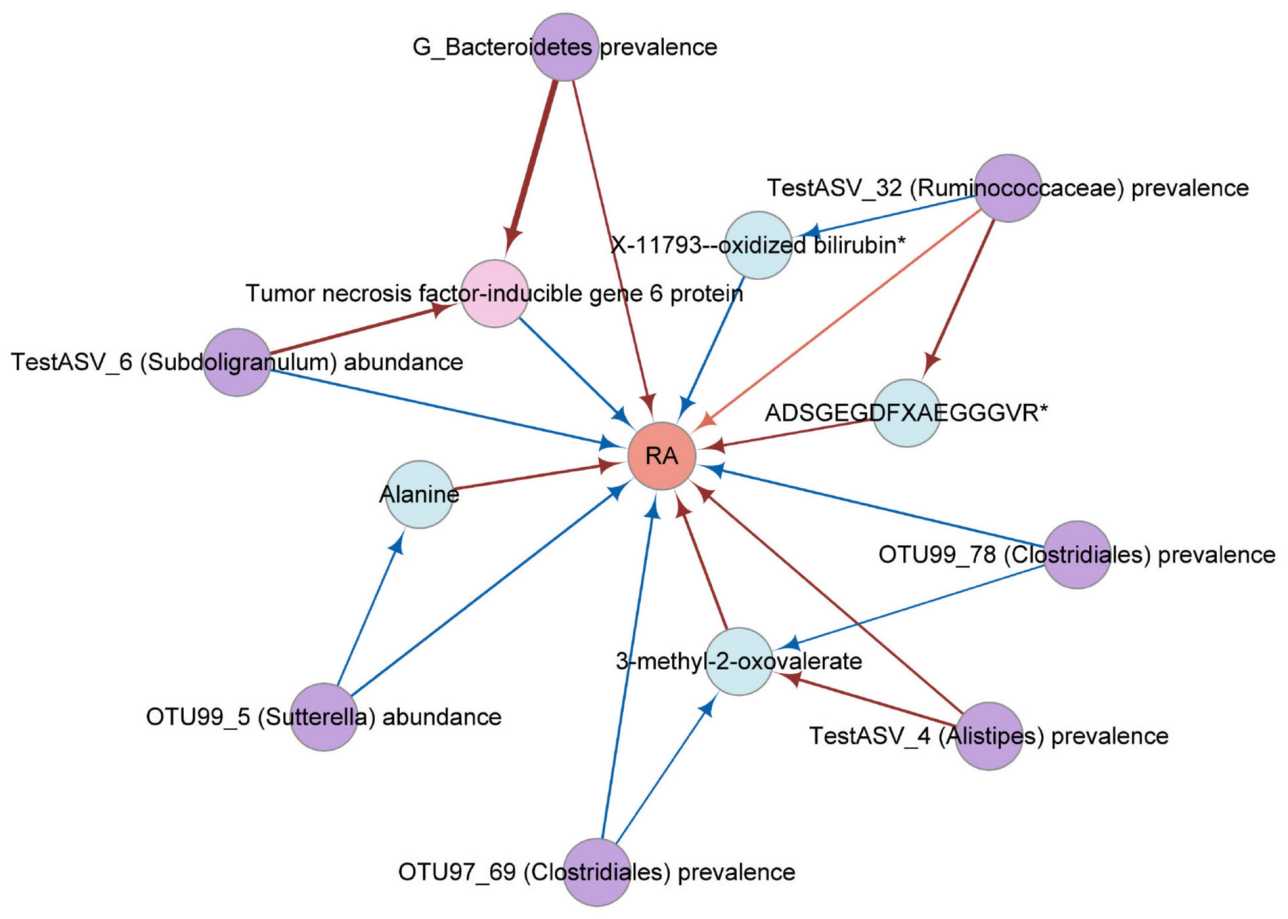
The network diagram from mediation analysis results. Each factor is significantly associated with other factor ( $P_{\text{BH}}<0.05$ ). Different colors were used to distinguish categories. Pink, cytokine; violet, gut microbial trait; orange, rheumatoid arthritis; blue, metabolite. The colors of the arrows represent different effects. Red is a risk factor for outcome (beta greater than 0); blue is a protective factor for outcome (beta less than 0). RA, rheumatoid arthritis; TSG-6, tumor necrosis factor-inducible gene 6 protein.

Give the limited evidence suggesting that microbiota has a causal effect on metabolites and cytokine (**Supplementary Table 9**). The Two-step MR and MVMR analyses identified that the effect of OTU99_78 (*Clostridiales*) prevalence (Proportion mediated (%): 58.22 and 48.70, respectively) and OTU97_69 (*Clostridiales*) prevalence (Proportion mediated (%): 58.07 and 52.89, respectively) had a mediated effect on RA through 3-methyl-2-oxovalerate. The Two-step MR analyses showed that the effect of OTU99_5 (*Sutterella*) abundance was partially mediated by alanine for RA (Proportion mediated (%): 21.10). There was evidence of an indirect effect of TestASV_4 (*Alistipes*) prevalence on RA via 3-methyl-2-oxovalerate (Proportion mediated (%): 24.73). Moreover, mediation analyses showed significant effects for TestASV_6 (*Subdoligranulum*) abundance (Proportion mediated (%): 7.75) and *G_Bacteroidetes* prevalence (Proportion mediated (%): 5.16) on TSG-6-mediated risk for RA (**Figure 4**; **Table 2**; **Supplementary Table 10**).

**Table 2:** Mediation analysis of the mediation effect of microbiota on RA via metabolite or cytokine.

Pathway	Exposure	Mediator	Total effect	Two-step MR		MVMR	
			Effect size	Effect size	Proportion mediated (%)	Effect size	Proportion mediated (%)
Microbiota→metablite→RA	OTU99_5 (Sutterella) abundance	Alanine	-6.89E-04	-1.45E-04	21.10	7.73E-05	NA
	TestASV_4 (Alistipes) prevalence	3-methyl-2-oxovalerate	6.76E-04	1.67E-04	24.73	-4.87E-06	NA
	OTU99_78 (Clostridiales) prevalence	3-methyl-2-oxovalerate	-4.92E-04	-2.86E-04	**58.22**	-2.40E-04	**48.70**
	OTU97_69 (Clostridiales) prevalence	3-methyl-2-oxovalerate	-4.93E-04	-2.86E-04	**58.07**	-2.61E-04	**52.89**
	TestASV_32 (Ruminococcaceae) prevalence	ADSGEGDFXAEGGGVR*	-2.45E-04	9.66E-06	NA	-5.50E-05	22.44
	TestASV_32 (Ruminococcaceae) prevalence	X-11793--oxidized bilirubin*	-2.45E-04	1.81E-05	NA	-8.68E-05	35.40
Microbiota→cytokine→RA	TestASV_6 (Subdoligranulum) abundance	TSG-6	-7.30E-04	-5.65E-05	7.75	1.23E-04	NA
	G_Bacteroidetes prevalence	TSG-6	4.60E-04	-4.50E-05	NA	2.37E-05	5.16

RA, rheumatoid arthritis; Total effect, the effect of the exposure on the risk of RA; MR, mendelian randomization; MVMR, multivariable mendelian randomization; Effect size, beta; TSG-6, tumor necrosis factor-inducible gene 6 protein. Bold, both the two-step Mendelian randomization and the multivariate Mendelian randomization analyses found the existence of this causal pathway. * is no special significance just part of the name of this metabolite.

While the combined power of the instruments in the MVMR analyses was considered strong and there was no evidence of heterogeneity in causal effect estimates (*F* > 9, Q_pval > 0.05) (**Table 2**; **Supplementary Table 11**), the indirect effect was estimated with lower precision compared to the two-step MR analysis. The test power of the product coefficient method and the difference coefficient method is essentially the same, but the standard error of the two methods differs, and the error probability of the difference coefficient method is higher than that of the coefficient product method.

### 3.5 Microbiota-Cytokine-RA causal associations predicted by MR analyses in human plasma experimentally validated *in vitro* cell experiments

**Figure 5:**
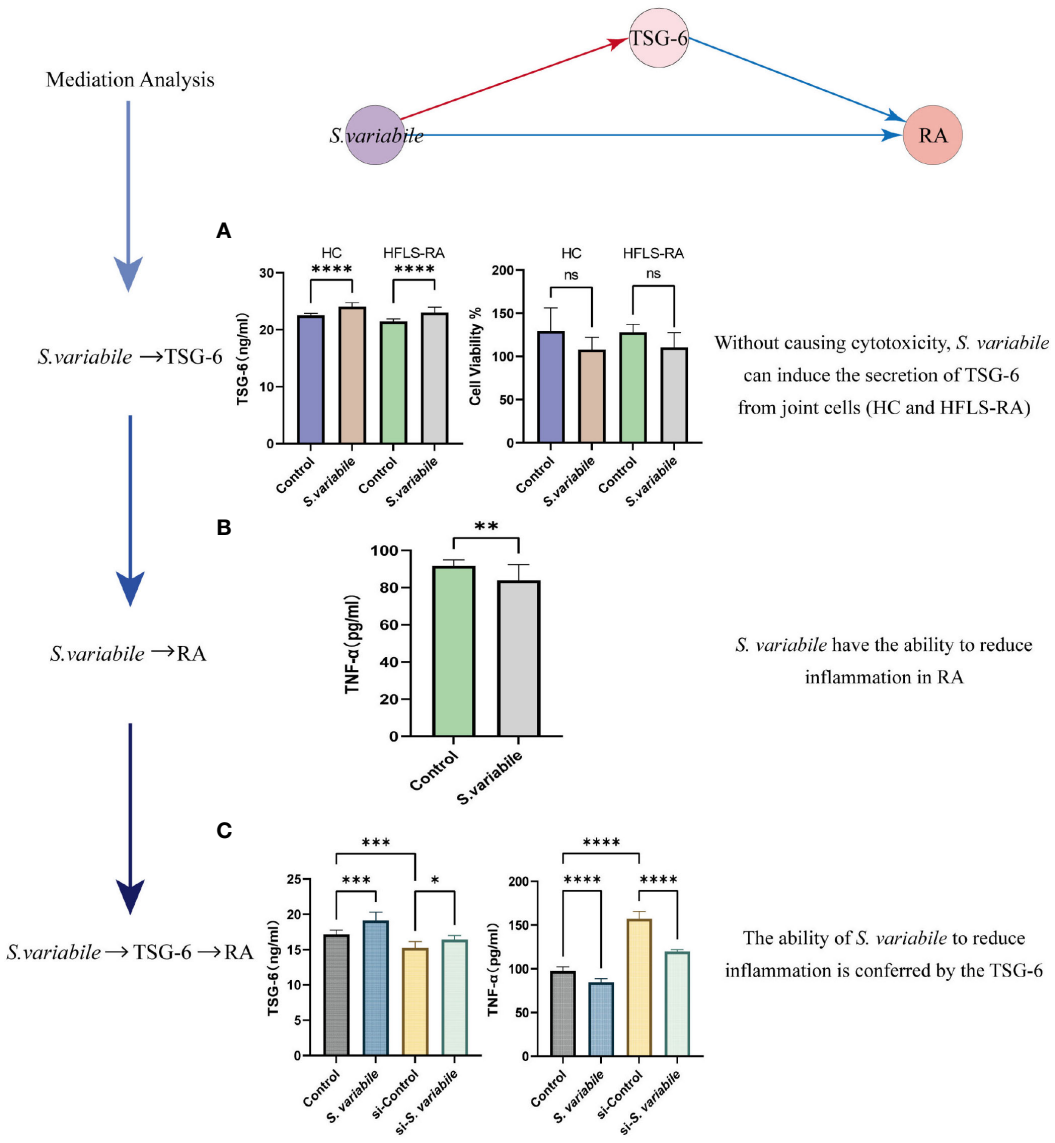
Validating the *S. variabile* - TSG-6 - RA pathway *in vitro*. **(A)** HC or HFLS-RA exposed to *S. variabile* or DMEM control (Left: secretion of TSG-6. Right: cytotoxicity assay). **(B)** TNF-α secretion in HFLS-RA exposed to *S. variabile* or DMEM control. **(C)** TSG-6 and TNF-α levels in the upper chamber of the transwell co-culture model (Control means untreated HC and HFLS-RA, *S. variabile* means co-culture of *S. variabile* with HC and HFLS-RA, si-Control means knockdown of TSG-6 in HC and HFLS-RA, si-*S. variabile* means the co-culture of *S. variabile* with TSG-6 gene silenced HC and HFLS-RA). RA, rheumatoid arthritis; TSG-6, tumor necrosis factor-inducible gene 6 protein; TNF-α, tumor necrosis factor-α; *S. variabile*, *Subdoligranulum variabile* DSM 15176^T^; HC, human chondrocytes; HFLS-RA, human rheumatoid arthritis fibroblast-like synoviocytes. Data are presented as the mean ± SEM (n = 3). *P< 0.05, **P< 0.01, ***P< 0.001, ****P< 0.0001, ns, no significance.

To demonstrate the credibility of the analytical results and the verifiability of the *in vitro* experiments, we chose a pathway in Microbiota-Cytokine-RA for experimental validation. TSG-6 was locally expressed at sites of inflammation and joint destruction, with the remarkable ability to significantly impede the progression of joint damage (44). So we initially established a co-culture of *S. variabile* with HC or RA to assess the mitigating effect of *S. variabile* on arthritis. We found that *S. variabile* can stimulate TSG-6 elevation and had almost no cytotoxic effects on the two cell lines at low doses (**Figure 5**).

To further verify the *S. variabile* - TSG-6 - RA causal associations, we established an *in vitro* model simulating the inflammatory microenvironment based on the optimal culture concentration and time determined from the co-culture experiments alone. Specifically, we co-cultured HC and HFLS-RA for a duration of 24 hours, then observed the secretion of TSG-6 and TNF-α in the upper chamber with and without the addition of *S. variabile*. TNF-α plays a central role in the regulation of RA-related molecules, it stimulating synovial fibroblasts to produce pro-inflammatory factorsis one of the features of RA (45). Our findings indicate that treatment with *S. variabile* resulted in a significant reduction in TNF-α expression levels and a significant increase in TSG-6 levels (**Figure 5**), when compared to the control group. To further confirm the involvement of TSG-6 in RA inflammation, siRNA was used to silence the TSG-6 gene. As expected, the reduction in TNF-α expression caused by *S. variabile*-treated HC was considerably reversed by knockdown of TSG-6.

**Figure 6:**
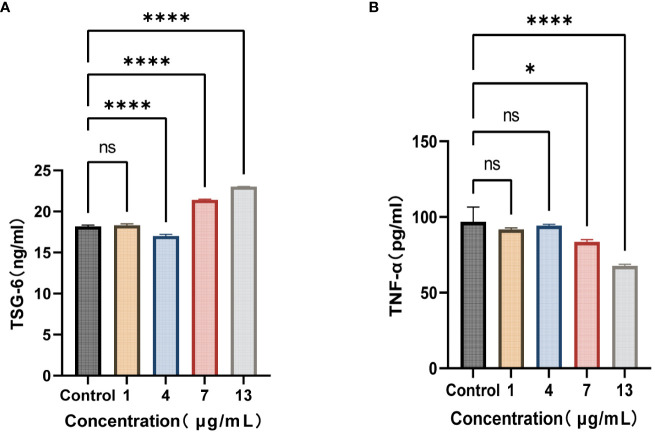
*S. variabile* derived exosomes relieve inflammation. **(A)** TSG-6 secretion from HFLS-RA at different exosome concentrations. **(B)** TNF-α secretion from HFLS-RA at different exosome concentrations. TSG-6, tumor necrosis factor-inducible gene 6 protein; TNF-α, tumor necrosis factor-α. Data are presented as the mean ± SEM (n = 3). *P< 0.05, ****P< 0.0001, ns, no significance.

All of the aforementioned experiments were conducted based on the assumption that *S. variabile* could be transferred from the gut to the joint. However, we did not find any evidence supporting the transfer of *S. variabile* through the Gut-Joint axis. Therefore, we hypothesized that the exosome derived from *S. variabile* can be effective in this process. In line with the co-culture alone, we co-incubated HFLS-RA with different concentrations of exosomes for 8 hours. Our results revealed that upon treatment with 7 µg/mL and 13 µg/mL of exosomes, the levels of TSG-6 in the treated group were significantly higher compared to the control group, resulting in subsequent reduction of TNF-α levels (**Figure 6**). These findings suggest that *S. variabile* and its exosome has the ability to diminish the levels of pro-inflammatory factors in the synovial tissue by upregulating TSG-6 expression, thereby substantiating its anti-inflammatory properties.

## 4 Discussion

To our knowledge, this is the first analysis to comprehensively reveal the causal relationship between the gut microbiota and RA through data analysis plus experimental validation. In the present MR study, we systematically assessed the causal relationship between gut microbiota characteristics and RA and further explored the causal pathways by which the gut microbiota influences RA. Our research revealed that 15 gut microbiota species significantly contribute to the pathogenesis of RA, and their influence on RA is partly mediated by 6 metabolites and 2 cytokines. We selected the *S. variabile* - TSG-6 - RA pathway and conducted preliminary validation using *in vitro* cell experiments. The results showed that *S. variabile* has the potential to promote TSG-6 secretion, thus reducing RA inflammation. So our study highlights the existence of a Gut-Metabolism/Cytokine-Joint axis.

An investigation uncovered a causal link between the gut microbiota composition and RA (46). Similarly, Guo et al. (47) documented specific gut bacterial taxa, such as *Methanobacteria* and *Alphaproteobacteria*, that were causally linked to the risk of RA. However, a separate study failed to observe such an association (4). The discrepancy in findings may be attributed to the use of GWAS datasets from distinct ethnic groups for RA and gut microbiota analysis, along with the stringent significance threshold employed in the study (*P* < 5×10^-8^), leading to a limited number of IVs. Our study differs from these investigations in three key aspects. Firstly, our study encompasses a more comprehensive range of exposures. In contrast to prior studies focusing solely on gut microbiotas, we also integrate metabolites and cytokines, evaluating their mediating relationship with the gut microbiota. This approach provides an opportunity to assess common factors causally linked to RA. To address the scarcity of available IVs, we applied a widely accepted threshold (*P* < 1×10^-5^) to identify eligible IVs (24). Additionally, we conducted a series of sensitivity analyses, including the MR Steiger test, Cochran Q statistic, and MR-PRESSO, to ensure result robustness. Furthermore, we performed cell experiments for validation. Notably, the causal associations identified in the discovery phase were further validated in experimental results, bolstering confidence in the true causal relationship.

In a previous study, we demonstrated the existence of the Gut-Joint axis through multiomics datasets (48), indicating that modifications in the gut microbiome may disrupt metabolic equilibrium, consequently exacerbating the advancement of early RA. In this work, we found that several genera in the gut microbiota, including *Sutterella* and *Subdoligranulum*, had suggestive protective effects against RA. Studies have shown an inverse relationship between *Sutterella* and the host inflammatory cytokine response (49). In addition, *Sutterella* does not appear to induce substantial inflammation, and it can limit intracellular bacterial species, including pathogenic bacteria such as *Fusobacterium* (50). These findings also suggest the potentially beneficial effects of *Sutterella*, which is consistent with the results of our study. Simultaneously, our investigation revealed that a significant proportion of the bacteria influencing the risk of RA were classified within the phylum *Firmicutes*. This finding aligns with the heightened presence of *Clostridiales*, a subset of *Firmicutes*, observed among RA patients in a cross-sectional study (51).

Rather than just concerning the causality between the gut microbiota and RA, we also considered the possible involvement of metabolites and cytokines in this process. It is worth noting that potentially beneficial microbes, such as *Sutterella* and *Clostridiales*, were found to be negatively correlated with alanine and 3-methyl-2-oxovaleric acid (a metabolic intermediate of valine). Both alanine and 3-methyl-2-oxovaleric acid have been reported to be correlated with intestinal permeability and the ability to regulate the intestinal barrier (52), and our results indicate that they may promote the progression of RA. Previous studies have shown that changes in the gut microbiota may influence inflammatory responses through changes in metabolic status, such as the metabolic pathway of amino acids including alanine and valine during antibiotic induction, which changes with changes in the gut flora, thereby controlling the progression of inflammation (53). These findings further confirm our conclusion that the gut microbiota can influence the progression of RA by altering metabolites.

For the results of the mediation analysis, we selected the *S. variabile* - TSG-6 - RA pathway for *in vitro* experimental validation. *Subdoligranulum* is a strictly anaerobic, butyrate-producing, gram-negative staining organism (54). We have chosen only one species, which has been isolated and cultured so far, for our *in vitro* experiments - the *Subdoligranulum variabile* DSM 15176^T^ strain. Indeed, a series of studies have demonstrated the probiotic effects of *S. variabile*, including the suppression of food allergies and amelioration of mucosal inflammation (55, 56). In the present study, *Subdoligranulum* was also shown to have a positive causal relationship with TSG-6. TSG-6 is considered to be a multifunctional protein with anti-inflammatory and tissue-protective properties. Some potential regulators of inflammatory processes in arthritis can influence the expression of TSG-6 RNA and contribute to the elevation of TSG-6 protein levels. The beneficial role of TSG-6 has been interpreted as inhibiting the association of toll-like receptors-2 (TLR2) with myeloid differentiation primary response protein 88 (MyD88), thereby suppressing nuclear factor kappa-B (NF-κB) activation, and prevented the expression of proinflammatory proteins (such as TNF-α) (45). In the experimental phase, we cultured *S. variabile* directly or indirectly with joint cells. The results demonstrated elevated expression of TSG-6 in joint cells such as HC and HFLS-RA after co-culturing with *S. variabile*, along with a downregulation of the disease indicator TNF-α in HFLS-RA. TNF-α, a key player in the pathogenesis of RA, is acknowledged for its pivotal role in inducing other proinflammatory mediators, such as IL-1β, chemokines, and proteases. Its involvement in instigating and sustaining inflammatory responses, as well as in the degradation of bone and cartilage, has been documented (57).

Recent studies have demonstrated that gut microbiota-derived exosomes can be detected in systemic tissues and play a key role in extraintestinal tissue diseases (58, 59). Exosomes are monolayer particles with 30–150 nm diameter. Their primary function lies in mediating intercellular communication and participating in a myriad of physiological processes, including inflammation, immune responses, cellular stress responses, and differentiation (60). Notably, exosomes derived from mesenchymal stem cells have been demonstrated significant therapeutic potential for RA (61). Next, we extracted exosomes from *S. variabile* and found their capability to stimulate the release of TSG-6, leading to a reduction in the expression of TNF-α. These findings reinforce the notion of an immunomodulatory role for TSG-6 in RA, while also highlighting the potential therapeutic value of *Subdoligranulum*.

However, our study has certain limitations. First, the relatively small sample size of the gut microbiota might slightly impact our estimations. To enhance accuracy, it is crucial to expand the sample size and evaluate the precise correlation between the gut microbiota and RA. Secondly, the analysis of bacterial taxa was limited to the genus level, rather than being conducted at more granular levels such as species or strain. Utilizing advanced shotgun metagenomic sequencing in microbiota GWASs can yield more precise and reliable results. Finally, while we successfully identified this significant gut microbiota, their validation has been limited to *in vitro* experiments, lacking animal models and human populations. Consequently, further investigations are warranted to elucidate their roles in the pathogenesis of RA.

## 5 Conclusion

In conclusion, we used studies with large sample sizes (RA, up to 463010 individuals) to illustrate that gut microbiota can influence inflammation by modifying amino acid metabolites and cytokines in patients with RA. Furthermore, we identified a pathway from the MR results that was most conducive to *in vitro* validation at the cellular level, thereby elucidating the role of *Subdoligranulum* and affirming the reliability of our analytical findings. To sum up, our findings support the potentially causal effects of the *Subdoligranulum* on RA.

## Data availability statement

The original contributions presented in the study are included in the article/**Supplementary Material**. Further inquiries can be directed to the corresponding authors.

## Author contributions

HL: Formal analysis, Investigation, Visualization, Writing – original draft. JD: Investigation, Validation, Visualization, Writing – original draft. CZ: Conceptualization, Supervision, Writing – original draft, Writing – review & editing. TH: Writing – review & editing. GZ: Writing – review & editing. QW: Writing – review & editing. LZ: Conceptualization, Funding acquisition, Project administration, Supervision, Writing – review & editing.
