# Supplementary material for: Gut Subdoligranulum variabile ameliorates rheumatoid arthritis by promoting TSG-6 synthesis from joint cells
Source: Front Immunol. 2024 Jun 10;15:1418717. doi: 10.3389/fimmu.2024.1418717 (PMC11229780; doi:10.3389/fimmu.2024.1418717)
Supplement: Supplementary file 1 [file DataSheet_1.docx]

**Contents**

**Figure S1.** KEGG enrichment analysis of the causal microbial taxa of rheumatoid arthritis.

**Figure S2.** Changes in TSG-6 content induced by *S. variabile*.

**Table S1.** The publicly available GWAS summary statistics of gut microbial traits, blood metabolites and immune factors.

**Table S2.** Mendelian randomization of gut microbiome on rheumatoid arthritis in European population (*P* < 1×10^-5^).

**Table S3.** Mendelian randomization of rheumatoid arthritis on microbial traits in European population.

**Table S4.** Mendelian randomization of metabolites and immune factors on rheumatoid arthritis in European population.

**Table S5.** Mendelian randomization of rheumatoid arthritis on immune factors in European population.

**Table S6.** The heterogeneity results from the Cochran's Q test and pleiotropy results from Egger intercept/MR-PROSSO analyses.

**Table S7.** Steiger test results from exposure to outcome.

**Table S8.** Colocalization analysis where loci provide evidence of a shared causal variant.

**Table S9.** Mendelian randomization of metabolites and immune factors on rheumatoid arthritis in European population.

**Table S10.** Two-step Mendelian randomization analysis of rheumatoid arthritis in European population.

**Table S11.** Multivariable Mendelian randomization analysis of rheumatoid arthritis in European population.

**Table S12.** Mendelian randomization of microbiota on rheumatoid arthritis (*P* < 5×10^-8^).

**Figure S1.** KEGG enrichment analysis of the causal microbial taxa of rheumatoid arthritis.


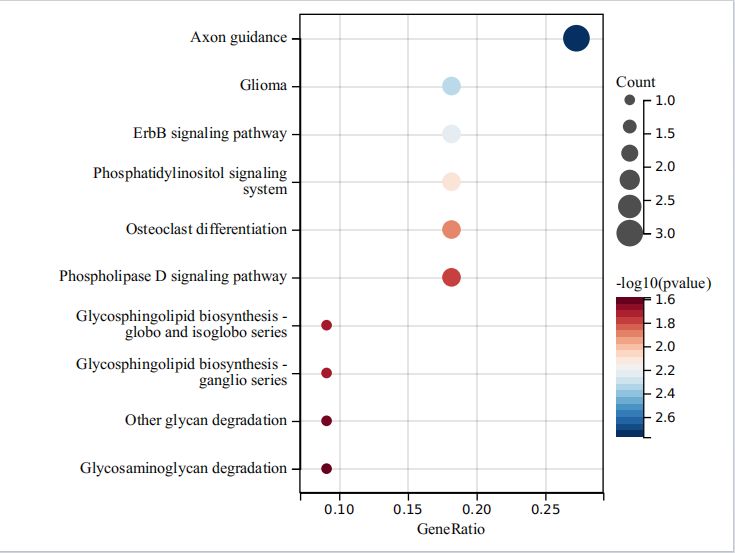


**Figure S2. Changes in TSG-6 content induced by S. variabile.**

**
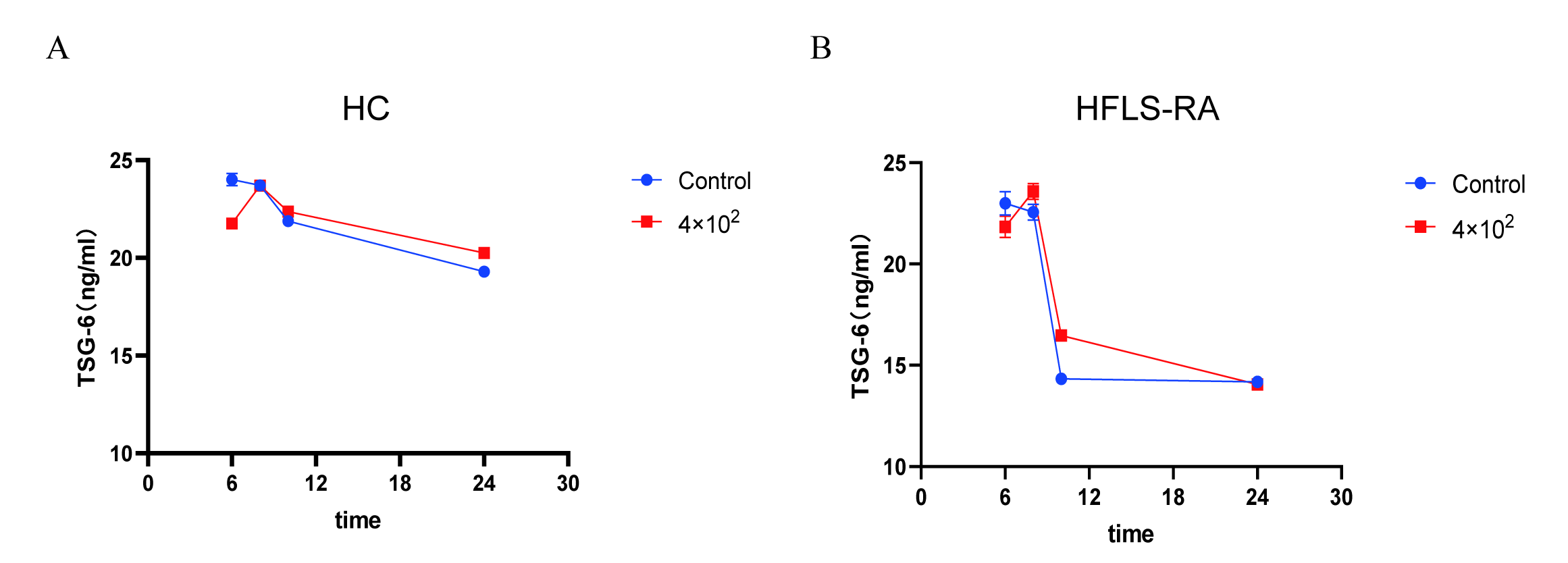
**

(A and B). The secretion of TSG-6 by HC (A) and HFLS-RA (B) over time in the presence or absence of *S. variabile*. TSG-6, tumor necrosis factor-inducible gene 6 protein; HC, human chondrocytes; HFLS-RA, human rheumatoid arthritis fibroblast-like synoviocytes.

**Table S1.** The publicly available GWAS summary statistics of gut microbial traits, blood metabolites and immune factors.

| ***Microbiomics*** |  | **Metabonomics** | | |  | **Immuneomics** | |  | |
| --- | --- | --- | --- | --- | --- | --- | --- | --- | --- |
| ***Trait*** |  | **Blood metabolite** | **Samplesize** | | | **Immune factor** | **Samplesize** | | |
| *C_Actinobacteria abundance* | *OTU99_34 (Holdemanella) abundance* | X-12510--2-aminooctanoic acid | | 7566 | | Tumor necrosis factor alpha levels | | | 3,454 |
| *C_Alphaproteobacteria abundance* | *OTU99_34 (Holdemanella) prevalence* | X-12100--hydroxytryptophan* | | 7499 | | Interleukin-6 levels | | | 8,189 |
| *C_Alphaproteobacteria prevalence* | *OTU99_35 (Ruminococcus) abundance* | X-11786--methylcysteine | | 2811 | | Tumor necrosis factor beta levels | | | 982 |
| *C_Bacteroidia abundance* | *OTU99_35 (Ruminococcus) prevalence* | X-11423--O-sulfo-L-tyrosine | | 7765 | | Tumor necrosis factor receptor superfamily member 9 levels | | | 982 |
| *C_Betaproteobacteria abundance* | *OTU99_39 (Alphaproteobacteria) abundance* | X-04499--3,4-dihydroxybutyrate | | 6948 | | Tumor necrosis factor ligand superfamily member 10 levels | | | 982 |
| *C_Clostridia abundance* | *OTU99_39 (Alphaproteobacteria) prevalence* | X-03056--N-[3-(2-Oxopyrrolidin-1-yl)propyl]acetamide | | 7812 | | Tumor necrosis factor receptor superfamily member 6 | | | 3,301 |
| *C_Erysipelotrichia abundance* | *OTU99_4 (Alistipes) abundance* | Valine | | 7808 | | Tumor necrosis factor ligand superfamily member 6, soluble form | | | 3,301 |
| *C_Gammaproteobacteria abundance* | *OTU99_4 (Alistipes) prevalence* | Urea | | 7796 | | Interleukin-20 | | | 3,301 |
| *C_Negativicutes abundance* | *OTU99_40 (Proteobacteria) abundance* | Tyrosine | | 7807 | | Interleukin-6 receptor subunit alpha | | | 3,301 |
| *C_Negativicutes prevalence* | *OTU99_40 (Proteobacteria) prevalence* | Tryptophan betaine | | 7439 | | Interleukin-6 receptor subunit beta | | | 3,301 |
| *F_Acidaminococcaceae abundance* | *OTU99_45 (Faecalibacterium) abundance* | Tryptophan | | 7804 | | Tumor necrosis factor receptor superfamily member 3 | | | 3,301 |
| *F_Acidaminococcaceae prevalence* | *OTU99_45 (Faecalibacterium) prevalence* | Trans-4-hydroxyproline | | 7802 | | Tumor necrosis factor receptor superfamily member 16 | | | 3,301 |
| *F_Bacteroidaceae abundance* | *OTU99_47 (Prevotella) abundance* | Threonine | | 6020 | | Tumor necrosis factor receptor superfamily member 19L | | | 3,301 |
| *F_Coriobacteriaceae abundance* | *OTU99_47 (Prevotella) prevalence* | Serotonin (5HT) | | 6139 | | Tumor necrosis factor | | | 3,301 |
| *F_Erysipelotrichaceae abundance* | *OTU99_5 (Sutterella) abundance* | Serine | | 7796 | | Complement C1q tumor necrosis factor-related protein 1 | | | 3,301 |
| *F_Lachnospiraceae abundance* | *OTU99_5 (Sutterella) prevalence* | Pyroglutamine* | | 7800 | | Tumor necrosis factor alpha-induced protein 3 | | | 3,301 |
| *F_Porphyromonadaceae abundance* | *OTU99_53 (Aestuariispira) abundance* | Proline | | 7816 | | Tumor necrosis factor-inducible gene 6 protein | | | 3,301 |
| *F_Prevotellaceae abundance* | *OTU99_53 (Aestuariispira) prevalence* | Pipecolate | | 7792 | | Tumor necrosis factor receptor superfamily member 10A | | | 3,301 |
| *F_Prevotellaceae prevalence* | *OTU99_54 (Holdemanella) abundance* | Phenyllactate (PLA) | | 6064 | | Tumor necrosis factor receptor superfamily member 10B | | | 3,301 |
| *F_Rikenellaceae abundance* | *OTU99_54 (Holdemanella) prevalence* | Phenylalanine | | 7803 | | Tumor necrosis factor receptor superfamily member 10D | | | 3,301 |
| *F_Ruminococcaceae abundance* | *OTU99_55 (Barnesiella) abundance* | Phenylacetylglutamine | | 7812 | | Tumor necrosis factor receptor superfamily member 11A | | | 3,301 |
| *F_Sutterellaceae abundance* | *OTU99_55 (Barnesiella) prevalence* | Phenylacetate | | 4754 | | Tumor necrosis factor receptor superfamily member 11B | | | 3,301 |
| *G_Alistipes abundance* | *OTU99_558 (Bacteroidales) abundance* | Phenol sulfate | | 7810 | | Complement C1q tumor necrosis factor-related protein 3 | | | 3,301 |
| *G_Alloprevotella abundance* | *OTU99_558 (Bacteroidales) prevalence* | P-cresol sulfate | | 7758 | | Tumor necrosis factor receptor superfamily member 12A | | | 3,301 |
| *G_Alloprevotella prevalence* | *OTU99_58 (Bacteroides) abundance* | Ornithine | | 7746 | | Tumor necrosis factor receptor superfamily member 13B | | | 3,301 |
| *G_Alphaproteobacteria abundance* | *OTU99_58 (Bacteroides) prevalence* | N-acetylthreonine | | 6909 | | Tumor necrosis factor receptor superfamily member 14 | | | 3,301 |
| *G_Alphaproteobacteria prevalence* | *OTU99_59 (Prevotella) abundance* | N-acetylornithine | | 7574 | | Tumor necrosis factor receptor superfamily member 17 | | | 3,301 |
| *G_Bacteroidales abundance* | *OTU99_59 (Prevotella) prevalence* | N-acetylglycine | | 7135 | | Tumor necrosis factor receptor superfamily member 18 | | | 3,301 |
| *G_Bacteroidales prevalence* | *OTU99_6 (Sutterella) abundance* | N-acetylalanine | | 7720 | | Tumor necrosis factor receptor superfamily member 19 | | | 3,301 |
| *G_Bacteroides abundance* | *OTU99_6 (Sutterella) prevalence* | Methionine | | 7795 | | Tumor necrosis factor receptor superfamily member 1A | | | 3,301 |
| *G_Bacteroidetes abundance* | *OTU99_62 (Ruminococcaceae) abundance* | Lysine | | 7812 | | Tumor necrosis factor receptor superfamily member 1B | | | 3,301 |
| *G_Bacteroidetes prevalence* | *OTU99_62 (Ruminococcaceae) prevalence* | Levulinate (4-oxovalerate) | | 6982 | | Complement C1q tumor necrosis factor-related protein 5 | | | 3,301 |
| *G_Barnesiella abundance* | *OTU99_65 (Sutterellaceae) abundance* | Leucine | | 7799 | | Tumor necrosis factor receptor superfamily member 21 | | | 3,301 |
| *G_Barnesiella prevalence* | *OTU99_65 (Sutterellaceae) prevalence* | Kynurenine | | 7816 | | Tumor necrosis factor receptor superfamily member 4 | | | 3,301 |
| *G_Catenibacterium abundance* | *OTU99_7 (Lachnospiraceae) abundance* | Isovalerylcarnitine | | 7789 | | Tumor necrosis factor receptor superfamily member 6B | | | 3,301 |
| *G_Catenibacterium prevalence* | *OTU99_7 (Lachnospiraceae) prevalence* | Isoleucine | | 7801 | | Tumor necrosis factor receptor superfamily member 8 | | | 3,301 |
| *G_Clostridiales abundance* | *OTU99_72 (Parabacteroides) abundance* | Isobutyrylcarnitine | | 7812 | | Tumor necrosis factor ligand superfamily member 11 | | | 3,301 |
| *G_Clostridium_XlVa abundance* | *OTU99_72 (Parabacteroides) prevalence* | Indolepropionate | | 7803 | | Tumor necrosis factor ligand superfamily member 12 | | | 3,301 |
| *G_Coprococcus abundance* | *OTU99_78 (Clostridiales) abundance* | Indolelactate | | 7378 | | Tumor necrosis factor ligand superfamily member 13B | | | 3,301 |
| *G_Coprococcus prevalence* | *OTU99_78 (Clostridiales) prevalence* | Indoleacetate | | 7618 | | Tumor necrosis factor ligand superfamily member 14 | | | 3,301 |
| *G_Faecalibacterium abundance* | *OTU99_8 (Ruminococcaceae) abundance* | Hydroxyisovaleroyl carnitine | | 5588 | | Tumor necrosis factor ligand superfamily member 15 | | | 3,301 |
| *G_Firmicutes abundance* | *OTU99_8 (Ruminococcaceae) prevalence* | Homocitrulline | | 4135 | | Complement C1q and tumor necrosis factor-related protein 9A | | | 3,301 |
| *G_Holdemanella abundance* | *OTU99_84 (Prevotella) abundance* | Histidine | | 7804 | | Tumor necrosis factor ligand superfamily member 18 | | | 3,301 |
| *G_Holdemanella prevalence* | *OTU99_84 (Prevotella) prevalence* | Glycine | | 7802 | | Tumor necrosis factor ligand superfamily member 4 | | | 3,301 |
| *G_Lachnospiraceae abundance* | *OTU99_85 (Alistipes) abundance* | Glutaroyl carnitine | | 7701 | | Tumor necrosis factor ligand superfamily member 8 | | | 3,301 |
| *G_Oscillibacter abundance* | *OTU99_85 (Alistipes) prevalence* | Glutamine | | 7821 | | Tumor necrosis factor ligand superfamily member 9 | | | 3,301 |
| *G_Parabacteroides abundance* | *OTU99_9 (Alistipes) abundance* | Glutamate | | 7804 | | Tumor necrosis factor receptor superfamily member 27 | | | 3,301 |
| *G_Parabacteroides prevalence* | *OTU99_9 (Alistipes) prevalence* | Dimethylarginine (SDMA + ADMA) | | 7347 | | Tumor necrosis factor receptor superfamily member EDAR | | | 3,301 |
| *G_Paraprevotella abundance* | *OTU99_92 (Ruminococcus) abundance* | Cysteine-glutathione disulfide | | 1997 | | TNF superfamily member 11 | | | 3,394 |
| *G_Paraprevotella prevalence* | *OTU99_92 (Ruminococcus) prevalence* | Cysteine | | 7692 | | TNF receptor superfamily member 10b | | | 3,394 |
| *G_Parasutterella abundance* | *OTU99_94 (Bacteroides) abundance* | Creatinine | | 7810 | | TNF superfamily member 10 | | | 3,394 |
| *G_Parasutterella prevalence* | *OTU99_94 (Bacteroides) prevalence* | Creatine | | 7822 | | TNF receptor superfamily member 1B | | | 3,394 |
| *G_Phascolarctobacterium abundance* | *OTU99_98 (Phascolarctobacterium) abundance* | Citrulline | | 7773 | |  | | |  |
| *G_Phascolarctobacterium prevalence* | *OTU99_98 (Phascolarctobacterium) prevalence* | C-glycosyltryptophan* | | 7786 | |  | | |  |
| *G_Porphyromonadaceae abundance* | *P_Actinobacteria abundance* | Betaine | | 7806 | |  | | |  |
| *G_Porphyromonadaceae prevalence* | *P_Bacteroidetes abundance* | Beta-hydroxyisovalerate | | 7283 | |  | | |  |
| *G_Prevotella abundance* | *P_Firmicutes abundance* | Aspartate | | 7721 | |  | | |  |
| *G_Prevotella prevalence* | *P_Proteobacteria abundance* | Asparagine | | 7761 | |  | | |  |
| *G_Prevotellaceae abundance* | *TestASV_1 (Bacteroides) abundance* | Arginine | | 7528 | |  | | |  |
| *G_Prevotellaceae prevalence* | *TestASV_1 (Bacteroides) prevalence* | Alpha-hydroxyisovalerate | | 7668 | |  | | |  |
| *G_Roseburia abundance* | *TestASV_10 (Parabacteroides) abundance* | Alanine | | 7788 | |  | | |  |
| *G_Ruminococcaceae abundance* | *TestASV_10 (Parabacteroides) prevalence* | 5-oxoproline | | 7802 | |  | | |  |
| *G_Ruminococcus abundance* | *TestASV_11 (Lachnospiraceae) abundance* | 4-methyl-2-oxopentanoate | | 7776 | |  | | |  |
| *G_Ruminococcus prevalence* | *TestASV_11 (Lachnospiraceae) prevalence* | 4-acetamidobutanoate | | 6930 | |  | | |  |
| *G_Ruminococcus2 abundance* | *TestASV_12 (Bacteroides) abundance* | 3-phenylpropionate (hydrocinnamate) | | 6182 | |  | | |  |
| *G_Subdoligranulum abundance* | *TestASV_12 (Bacteroides) prevalence* | 3-methylhistidine | | 5885 | |  | | |  |
| *G_Sutterella abundance* | *TestASV_13 (Bacteroides) abundance* | 3-methyl-2-oxovalerate | | 7779 | |  | | |  |
| *G_Sutterella prevalence* | *TestASV_13 (Bacteroides) prevalence* | 3-methyl-2-oxobutyrate | | 7648 | |  | | |  |
| *O_Bacteroidales abundance* | *TestASV_14 (Lachnospiraceae) abundance* | 3-methoxytyrosine | | 5997 | |  | | |  |
| *O_Burkholderiales abundance* | *TestASV_14 (Lachnospiraceae) prevalence* | 3-indoxyl sulfate | | 7787 | |  | | |  |
| *O_Clostridiales abundance* | *TestASV_15 (Bacteroides) abundance* | 3-(4-hydroxyphenyl)lactate | | 7795 | |  | | |  |
| *O_Coriobacteriales abundance* | *TestASV_15 (Bacteroides) prevalence* | 3-(3-hydroxyphenyl)propionate | | 1163 | |  | | |  |
| *O_Erysipelotrichales abundance* | *TestASV_16 (Bacteroides) abundance* | 2-methylbutyroylcarnitine | | 7420 | |  | | |  |
| *O_Selenomonadales abundance* | *TestASV_16 (Bacteroides) prevalence* | 2-hydroxyisobutyrate | | 6539 | |  | | |  |
| *O_Selenomonadales prevalence* | *TestASV_17 (Bacteroides) abundance* | 2-hydroxybutyrate (AHB) | | 7815 | |  | | |  |
| *OTU97_1 (Bacteroides) abundance* | *TestASV_17 (Bacteroides) prevalence* | 2-aminobutyrate | | 7814 | |  | | |  |
| *OTU97_1 (Bacteroides) prevalence* | *TestASV_18 (Prevotella) abundance* | Threitol | | 7381 | |  | | |  |
| *OTU97_100 (Ruminococcaceae) abundance* | *TestASV_18 (Prevotella) prevalence* | Pyruvate | | 7682 | |  | | |  |
| *OTU97_100 (Ruminococcaceae) prevalence* | *TestASV_19 (Prevotella) abundance* | Mannose | | 7793 | |  | | |  |
| *OTU97_101 (Sutterella) abundance* | *TestASV_19 (Prevotella) prevalence* | Mannitol | | 5917 | |  | | |  |
| *OTU97_101 (Sutterella) prevalence* | *TestASV_2 (Bacteroides) abundance* | Lactate | | 7814 | |  | | |  |
| *OTU97_102 (Prevotella) abundance* | *TestASV_2 (Bacteroides) prevalence* | Glycerate | | 7778 | |  | | |  |
| *OTU97_102 (Prevotella) prevalence* | *TestASV_20 (Phascolarctobacterium) abundance* | Glucose | | 7773 | |  | | |  |
| *OTU97_105 (Ruminococcaceae) abundance* | *TestASV_20 (Phascolarctobacterium) prevalence* | Fructose | | 7781 | |  | | |  |
| *OTU97_105 (Ruminococcaceae) prevalence* | *TestASV_21 (Ruminococcaceae) abundance* | Erythrose | | 7263 | |  | | |  |
| *OTU97_106 (Ruminococcaceae) abundance* | *TestASV_21 (Ruminococcaceae) prevalence* | Erythronate* | | 7752 | |  | | |  |
| *OTU97_106 (Ruminococcaceae) prevalence* | *TestASV_22 (Sutterella) abundance* | Arabinose | | 5889 | |  | | |  |
| *OTU97_108 (Phascolarctobacterium) abundance* | *TestASV_22 (Sutterella) prevalence* | 1,6-anhydroglucose | | 3663 | |  | | |  |
| *OTU97_108 (Phascolarctobacterium) prevalence* | *TestASV_23 (Barnesiella) abundance* | 1,5-anhydroglucitol (1,5-AG) | | 7746 | |  | | |  |
| *OTU97_109 (Paraprevotella) abundance* | *TestASV_23 (Barnesiella) prevalence* | X-11793--oxidized bilirubin* | | 7611 | |  | | |  |
| *OTU97_109 (Paraprevotella) prevalence* | *TestASV_24 (Bacteroides) abundance* | X-11593--O-methylascorbate* | | 7788 | |  | | |  |
| *OTU97_11 (Parabacteroides) abundance* | *TestASV_24 (Bacteroides) prevalence* | Threonate | | 7782 | |  | | |  |
| *OTU97_11 (Parabacteroides) prevalence* | *TestASV_25 (Sutterella) abundance* | Pyridoxate | | 7703 | |  | | |  |
| *OTU97_113 (Bacteroidales) abundance* | *TestASV_25 (Sutterella) prevalence* | Pantothenate | | 7604 | |  | | |  |
| *OTU97_113 (Bacteroidales) prevalence* | *TestASV_26 (Phascolarctobacterium) abundance* | Heme* | | 6380 | |  | | |  |
| *OTU97_117 (Ruminococcus) abundance* | *TestASV_26 (Phascolarctobacterium) prevalence* | Gamma-tocopherol | | 6226 | |  | | |  |
| *OTU97_117 (Ruminococcus) prevalence* | *TestASV_27 (Ruminococcaceae) abundance* | Biliverdin | | 6686 | |  | | |  |
| *OTU97_119 (Desulfovibrio) abundance* | *TestASV_27 (Ruminococcaceae) prevalence* | Bilirubin (Z,Z) | | 6812 | |  | | |  |
| *OTU97_119 (Desulfovibrio) prevalence* | *TestASV_28 (Holdemanella) abundance* | Bilirubin (E,Z or Z,E)* | | 5295 | |  | | |  |
| *OTU97_12 (Bacteroides) abundance* | *TestASV_28 (Holdemanella) prevalence* | Bilirubin (E,E)* | | 7748 | |  | | |  |
| *OTU97_12 (Bacteroides) prevalence* | *TestASV_29 (Barnesiella) abundance* | Ascorbate (Vitamin C) | | 2085 | |  | | |  |
| *OTU97_126 (Prevotella) abundance* | *TestASV_29 (Barnesiella) prevalence* | Alpha-tocopherol | | 7725 | |  | | |  |
| *OTU97_126 (Prevotella) prevalence* | *TestASV_3 (Bacteroides) abundance* | Succinylcarnitine | | 6948 | |  | | |  |
| *OTU97_13 (Subdoligranulum) abundance* | *TestASV_3 (Bacteroides) prevalence* | Phosphate | | 7789 | |  | | |  |
| *OTU97_13 (Subdoligranulum) prevalence* | *TestASV_30 (Paraprevotella) abundance* | Malate | | 7384 | |  | | |  |
| *OTU97_130 (Butyrivibrio) abundance* | *TestASV_30 (Paraprevotella) prevalence* | Citrate | | 7813 | |  | | |  |
| *OTU97_130 (Butyrivibrio) prevalence* | *TestASV_31 (Ruminococcus) abundance* | Alpha-ketoglutarate | | 6130 | |  | | |  |
| *OTU97_137 (Catenibacterium) abundance* | *TestASV_31 (Ruminococcus) prevalence* | Acetylphosphate | | 7789 | |  | | |  |
| *OTU97_137 (Catenibacterium) prevalence* | *TestASV_32 (Ruminococcaceae) abundance* | X-12990--docosapentaenoic acid (n6-DPA) | | 2581 | |  | | |  |
| *OTU97_138 (Oscillibacter) abundance* | *TestASV_32 (Ruminococcaceae) prevalence* | Undecanoate (11:0) | | 7500 | |  | | |  |
| *OTU97_138 (Oscillibacter) prevalence* | *TestASV_33 (Paraprevotella) abundance* | Stearidonate (18:4n3) | | 7775 | |  | | |  |
| *OTU97_140 (Bacteroides) abundance* | *TestASV_33 (Paraprevotella) prevalence* | Stearate (18:0) | | 7803 | |  | | |  |
| *OTU97_140 (Bacteroides) prevalence* | *TestASV_34 (Phascolarctobacterium) abundance* | Pentadecanoate (15:0) | | 7502 | |  | | |  |
| *OTU97_15 (Parasutterella) abundance* | *TestASV_34 (Phascolarctobacterium) prevalence* | Pelargonate (9:0) | | 7803 | |  | | |  |
| *OTU97_15 (Parasutterella) prevalence* | *TestASV_35 (Alphaproteobacteria) abundance* | Palmitoleate (16:1n7) | | 7776 | |  | | |  |
| *OTU97_150 (Bacteroides) abundance* | *TestASV_35 (Alphaproteobacteria) prevalence* | Palmitate (16:0) | | 7800 | |  | | |  |
| *OTU97_150 (Bacteroides) prevalence* | *TestASV_36 (Aestuariispira) abundance* | Oleate (18:1n9) | | 7768 | |  | | |  |
| *OTU97_16 (Faecalibacterium) abundance* | *TestASV_36 (Aestuariispira) prevalence* | Nonadecanoate (19:0) | | 7783 | |  | | |  |
| *OTU97_16 (Faecalibacterium) prevalence* | *TestASV_37 (Ruminococcaceae) abundance* | Myristoleate (14:1n5) | | 7804 | |  | | |  |
| *OTU97_163 (Bacteroides) abundance* | *TestASV_37 (Ruminococcaceae) prevalence* | Myristate (14:0) | | 7811 | |  | | |  |
| *OTU97_163 (Bacteroides) prevalence* | *TestASV_38 (Ruminococcus) abundance* | Margarate (17:0) | | 7796 | |  | | |  |
| *OTU97_165 (Porphyromonadaceae) abundance* | *TestASV_38 (Ruminococcus) prevalence* | Linolenate [alpha or gamma; (18:3n3 or 6)] | | 7786 | |  | | |  |
| *OTU97_165 (Porphyromonadaceae) prevalence* | *TestASV_39 (Bacteroides) abundance* | Linoleate (18:2n6) | | 7780 | |  | | |  |
| *OTU97_17 (Parabacteroides) abundance* | *TestASV_39 (Bacteroides) prevalence* | Laurate (12:0) | | 7793 | |  | | |  |
| *OTU97_17 (Parabacteroides) prevalence* | *TestASV_4 (Alistipes) abundance* | Heptanoate (7:0) | | 7802 | |  | | |  |
| *OTU97_173 (Bacteroides) abundance* | *TestASV_4 (Alistipes) prevalence* | Eicosenoate (20:1n9 or 11) | | 7799 | |  | | |  |
| *OTU97_173 (Bacteroides) prevalence* | *TestASV_40 (Barnesiella) prevalence* | Eicosapentaenoate (EPA; 20:5n3) | | 7816 | |  | | |  |
| *OTU97_19 (Bacteria) abundance* | *TestASV_41 (Barnesiella) abundance* | Docosapentaenoate (n3 DPA; 22:5n3) | | 7821 | |  | | |  |
| *OTU97_19 (Bacteria) prevalence* | *TestASV_41 (Barnesiella) prevalence* | Docosahexaenoate (DHA; 22:6n3) | | 7818 | |  | | |  |
| *OTU97_2 (Bacteroides) abundance* | *TestASV_42 (Alphaproteobacteria) abundance* | Dihomo-linolenate (20:3n3 or n6) | | 7805 | |  | | |  |
| *OTU97_2 (Bacteroides) prevalence* | *TestASV_42 (Alphaproteobacteria) prevalence* | Dihomo-linoleate (20:2n6) | | 7800 | |  | | |  |
| *OTU97_21 (Parasutterella) abundance* | *TestASV_43 (Parasutterella) abundance* | Caprylate (8:0) | | 7802 | |  | | |  |
| *OTU97_21 (Parasutterella) prevalence* | *TestASV_43 (Parasutterella) prevalence* | Caproate (6:0) | | 7811 | |  | | |  |
| *OTU97_23 (Faecalibacterium) abundance* | *TestASV_44 (Parasutterella) abundance* | Arachidonate (20:4n6) | | 7816 | |  | | |  |
| *OTU97_23 (Faecalibacterium) prevalence* | *TestASV_44 (Parasutterella) prevalence* | Adrenate (22:4n6) | | 7778 | |  | | |  |
| *OTU97_235 (Phascolarctobacterium) abundance* | *TestASV_45 (Clostridiales) abundance* | 5-dodecenoate (12:1n7) | | 7770 | |  | | |  |
| *OTU97_235 (Phascolarctobacterium) prevalence* | *TestASV_45 (Clostridiales) prevalence* | 10-undecenoate (11:1n1) | | 7806 | |  | | |  |
| *OTU97_24 (Prevotella) abundance* | *TestASV_46 (Sutterella) abundance* | 10-nonadecenoate (19:1n9) | | 7789 | |  | | |  |
| *OTU97_24 (Prevotella) prevalence* | *TestASV_46 (Sutterella) prevalence* | 10-heptadecenoate (17:1n7) | | 7795 | |  | | |  |
| *OTU97_257 (Mitsuokella) abundance* | *TestASV_47 (Parasutterella) abundance* | X-13431--nonanoylcarnitine* | | 6591 | |  | | |  |
| *OTU97_257 (Mitsuokella) prevalence* | *TestASV_47 (Parasutterella) prevalence* | X-13183--stearamide | | 2503 | |  | | |  |
| *OTU97_26 (Parasutterella) abundance* | *TestASV_48 (Sutterella) abundance* | X-12442--5,8-tetradecadienoate | | 7780 | |  | | |  |
| *OTU97_26 (Parasutterella) prevalence* | *TestASV_48 (Sutterella) prevalence* | X-12441--12-hydroxyeicosatetraenoate (12-HETE) | | 2761 | |  | | |  |
| *OTU97_27 (Bacteroides) abundance* | *TestASV_49 (Parasutterella) abundance* | X-11445--5-alpha-pregnan-3beta,20alpha-disulfate | | 2570 | |  | | |  |
| *OTU97_27 (Bacteroides) prevalence* | *TestASV_49 (Parasutterella) prevalence* | Valerate | | 4102 | |  | | |  |
| *OTU97_277 (Prevotella) abundance* | *TestASV_5 (Bacteroides) abundance* | Ursodeoxycholate | | 5477 | |  | | |  |
| *OTU97_277 (Prevotella) prevalence* | *TestASV_5 (Bacteroides) prevalence* | Tetradecanedioate | | 6046 | |  | | |  |
| *OTU97_3 (Bacteroides) abundance* | *TestASV_6 (Subdoligranulum) abundance* | Taurolithocholate 3-sulfate | | 6904 | |  | | |  |
| *OTU97_3 (Bacteroides) prevalence* | *TestASV_6 (Subdoligranulum) prevalence* | Taurodeoxycholate | | 1564 | |  | | |  |
| *OTU97_30 (Roseburia) abundance* | *TestASV_7 (Bacteroides) abundance* | Taurocholate | | 4032 | |  | | |  |
| *OTU97_30 (Roseburia) prevalence* | *TestASV_7 (Bacteroides) prevalence* | Taurochenodeoxycholate | | 5716 | |  | | |  |
| *OTU97_31 (Prevotella) abundance* | *TestASV_8 (Bacteroides) abundance* | Stearoylcarnitine | | 7183 | |  | | |  |
| *OTU97_31 (Prevotella) prevalence* | *TestASV_8 (Bacteroides) prevalence* | Scyllo-inositol | | 6500 | |  | | |  |
| *OTU97_33 (Holdemanella) abundance* | *TestASV_9 (Alistipes) abundance* | Propionylcarnitine | | 7813 | |  | | |  |
| *OTU97_33 (Holdemanella) prevalence* | *TestASV_9 (Alistipes) prevalence* | Palmitoylcarnitine | | 7701 | |  | | |  |
| *OTU97_34 (Ruminococcus) abundance* |  | Palmitoyl sphingomyelin | | 7814 | |  | | |  |
| *OTU97_34 (Ruminococcus) prevalence* |  | Oleoylcarnitine | | 7708 | |  | | |  |
| *OTU97_354 (Alloprevotella) abundance* |  | Octanoylcarnitine | | 7790 | |  | | |  |
| *OTU97_354 (Alloprevotella) prevalence* |  | Octadecanedioate | | 7300 | |  | | |  |
| *OTU97_38 (Alphaproteobacteria) abundance* |  | N-Butyl Oleate | | 4542 | |  | | |  |
| *OTU97_38 (Alphaproteobacteria) prevalence* |  | Myo-inositol | | 7803 | |  | | |  |
| *OTU97_39 (Proteobacteria) abundance* |  | Laurylcarnitine | | 5170 | |  | | |  |
| *OTU97_39 (Proteobacteria) prevalence* |  | Lathosterol | | 5457 | |  | | |  |
| *OTU97_4 (Alistipes) abundance* |  | Isovalerate | | 7080 | |  | | |  |
| *OTU97_4 (Alistipes) prevalence* |  | Hyodeoxycholate | | 6025 | |  | | |  |
| *OTU97_46 (Lachnospiraceae) abundance* |  | Hexanoylcarnitine | | 7786 | |  | | |  |
| *OTU97_46 (Lachnospiraceae) prevalence* |  | Hexadecanedioate | | 6887 | |  | | |  |
| *OTU97_5 (Sutterella) abundance* |  | Glycodeoxycholate | | 1477 | |  | | |  |
| *OTU97_5 (Sutterella) prevalence* |  | Glycocholate | | 5995 | |  | | |  |
| *OTU97_50 (Aestuariispira) abundance* |  | Glycochenodeoxycholate | | 7087 | |  | | |  |
| *OTU97_50 (Aestuariispira) prevalence* |  | Glycerophosphorylcholine (GPC) | | 7156 | |  | | |  |
| *OTU97_51 (Barnesiella) abundance* |  | Glycerol 3-phosphate (G3P) | | 7781 | |  | | |  |
| *OTU97_51 (Barnesiella) prevalence* |  | Glycerol | | 7800 | |  | | |  |
| *OTU97_53 (Bacteroides) abundance* |  | Estrone 3-sulfate | | 957 | |  | | |  |
| *OTU97_53 (Bacteroides) prevalence* |  | Epiandrosterone sulfate | | 7769 | |  | | |  |
| *OTU97_56 (Ruminococcaceae) abundance* |  | Dodecanedioate | | 6478 | |  | | |  |
| *OTU97_56 (Ruminococcaceae) prevalence* |  | Deoxycholate | | 5194 | |  | | |  |
| *OTU97_58 (Sutterellaceae) abundance* |  | Dehydroisoandrosterone sulfate (DHEA-S) | | 7793 | |  | | |  |
| *OTU97_58 (Sutterellaceae) prevalence* |  | Decanoylcarnitine | | 7766 | |  | | |  |
| *OTU97_6 (Sutterella) abundance* |  | Cortisone | | 7575 | |  | | |  |
| *OTU97_6 (Sutterella) prevalence* |  | Cortisol | | 7795 | |  | | |  |
| *OTU97_65 (Parabacteroides) abundance* |  | Cis-4-decenoyl carnitine | | 7660 | |  | | |  |
| *OTU97_65 (Parabacteroides) prevalence* |  | Choline | | 7750 | |  | | |  |
| *OTU97_69 (Clostridiales) abundance* |  | Cholesterol | | 7813 | |  | | |  |
| *OTU97_69 (Clostridiales) prevalence* |  | Cholate | | 5929 | |  | | |  |
| *OTU97_7 (Lachnospiraceae) abundance* |  | Chiro-inositol | | 2729 | |  | | |  |
| *OTU97_7 (Lachnospiraceae) prevalence* |  | Carnitine | | 7797 | |  | | |  |
| *OTU97_73 (Prevotella) abundance* |  | Butyrylcarnitine | | 7796 | |  | | |  |
| *OTU97_73 (Prevotella) prevalence* |  | Androsterone sulfate | | 7785 | |  | | |  |
| *OTU97_74 (Alistipes) abundance* |  | Acetylcarnitine | | 7805 | |  | | |  |
| *OTU97_74 (Alistipes) prevalence* |  | 7-alpha-hydroxy-3-oxo-4-cholestenoate (7-Hoca) | | 7784 | |  | | |  |
| *OTU97_8 (Ruminococcaceae) abundance* |  | 5alpha-androstan-3beta,17beta-diol disulfate | | 7345 | |  | | |  |
| *OTU97_8 (Ruminococcaceae) prevalence* |  | 4-androsten-3beta,17beta-diol disulfate 2* | | 7776 | |  | | |  |
| *OTU97_80 (Ruminococcus) abundance* |  | 4-androsten-3beta,17beta-diol disulfate 1* | | 7804 | |  | | |  |
| *OTU97_80 (Ruminococcus) prevalence* |  | 3-hydroxybutyrate (BHBA) | | 7820 | |  | | |  |
| *OTU97_82 (Bacteroides) abundance* |  | 3-dehydrocarnitine* | | 7809 | |  | | |  |
| *OTU97_82 (Bacteroides) prevalence* |  | 3-carboxy-4-methyl-5-propyl-2-furanpropanoate (CMPF) | | 7812 | |  | | |  |
| *OTU97_86 (Phascolarctobacterium) abundance* |  | 2-tetradecenoyl carnitine | | 6998 | |  | | |  |
| *OTU97_86 (Phascolarctobacterium) prevalence* |  | 2-stearoylglycerophosphocholine* | | 7730 | |  | | |  |
| *OTU97_9 (Alistipes) abundance* |  | 2-palmitoylglycerophosphocholine* | | 7695 | |  | | |  |
| *OTU97_9 (Alistipes) prevalence* |  | 2-oleoylglycerophosphocholine* | | 7524 | |  | | |  |
| *OTU97_95 (Cytophagales) abundance* |  | 2-linoleoylglycerophosphocholine* | | 6894 | |  | | |  |
| *OTU97_95 (Cytophagales) prevalence* |  | 2-hydroxystearate | | 7763 | |  | | |  |
| *OTU97_97 (Clostridiales) abundance* |  | 2-hydroxypalmitate | | 7797 | |  | | |  |
| *OTU97_97 (Clostridiales) prevalence* |  | 2-hydroxyglutarate | | 6287 | |  | | |  |
| *OTU99_1 (Bacteroides) abundance* |  | 1-stearoylglycerophosphoinositol | | 7694 | |  | | |  |
| *OTU99_1 (Bacteroides) prevalence* |  | 1-stearoylglycerophosphoethanolamine | | 7369 | |  | | |  |
| *OTU99_106 (Barnesiella) abundance* |  | 1-stearoylglycerophosphocholine | | 7817 | |  | | |  |
| *OTU99_106 (Barnesiella) prevalence* |  | 1-stearoylglycerol (1-monostearin) | | 6965 | |  | | |  |
| *OTU99_108 (Cytophagales) abundance* |  | 1-palmitoylglycerophosphoinositol* | | 6282 | |  | | |  |
| *OTU99_108 (Cytophagales) prevalence* |  | 1-palmitoylglycerophosphoethanolamine | | 7763 | |  | | |  |
| *OTU99_11 (Parabacteroides) abundance* |  | 1-palmitoylglycerophosphocholine | | 7803 | |  | | |  |
| *OTU99_11 (Parabacteroides) prevalence* |  | 1-palmitoylglycerol (1-monopalmitin) | | 7429 | |  | | |  |
| *OTU99_110 (Clostridiales) abundance* |  | 1-palmitoleoylglycerophosphocholine* | | 7812 | |  | | |  |
| *OTU99_110 (Clostridiales) prevalence* |  | 1-oleoylglycerophosphoethanolamine | | 7749 | |  | | |  |
| *OTU99_116 (Sutterella) abundance* |  | 1-oleoylglycerophosphocholine | | 7812 | |  | | |  |
| *OTU99_116 (Sutterella) prevalence* |  | 1-oleoylglycerol (1-monoolein) | | 5717 | |  | | |  |
| *OTU99_12 (Bacteroides) abundance* |  | 1-myristoylglycerophosphocholine | | 7812 | |  | | |  |
| *OTU99_12 (Bacteroides) prevalence* |  | 1-linoleoylglycerophosphoethanolamine* | | 7817 | |  | | |  |
| *OTU99_120 (Ruminococcaceae) abundance* |  | 1-linoleoylglycerophosphocholine | | 7795 | |  | | |  |
| *OTU99_120 (Ruminococcaceae) prevalence* |  | 1-linoleoylglycerol (1-monolinolein) | | 2797 | |  | | |  |
| *OTU99_121 (Ruminococcaceae) abundance* |  | 1-heptadecanoylglycerophosphocholine | | 7422 | |  | | |  |
| *OTU99_121 (Ruminococcaceae) prevalence* |  | 1-eicosatrienoylglycerophosphocholine* | | 7809 | |  | | |  |
| *OTU99_123 (Phascolarctobacterium) abundance* |  | 1-eicosadienoylglycerophosphocholine* | | 6892 | |  | | |  |
| *OTU99_123 (Phascolarctobacterium) prevalence* |  | 1-docosahexaenoylglycerophosphocholine* | | 7798 | |  | | |  |
| *OTU99_124 (Paraprevotella) abundance* |  | 1-arachidonoylglycerophosphoinositol* | | 7797 | |  | | |  |
| *OTU99_124 (Paraprevotella) prevalence* |  | 1-arachidonoylglycerophosphoethanolamine* | | 7798 | |  | | |  |
| *OTU99_13 (Subdoligranulum) abundance* |  | 1-arachidonoylglycerophosphocholine* | | 7507 | |  | | |  |
| *OTU99_13 (Subdoligranulum) prevalence* |  | 15-methylpalmitate (isobar with 2-methylpalmitate) | | 7371 | |  | | |  |
| *OTU99_132 (Bacteroidales) abundance* |  | Xanthine | | 6845 | |  | | |  |
| *OTU99_132 (Bacteroidales) prevalence* |  | X-12095--N1-methyl-3-pyridone-4-carboxamide | | 7711 | |  | | |  |
| *OTU99_137 (Ruminococcus) abundance* |  | X-11422--xanthine | | 6346 | |  | | |  |
| *OTU99_137 (Ruminococcus) prevalence* |  | Uridine | | 7800 | |  | | |  |
| *OTU99_140 (Desulfovibrio) abundance* |  | Urate | | 7819 | |  | | |  |
| *OTU99_140 (Desulfovibrio) prevalence* |  | Pseudouridine | | 7785 | |  | | |  |
| *OTU99_150 (Prevotella) abundance* |  | N2,N2-dimethylguanosine | | 5228 | |  | | |  |
| *OTU99_150 (Prevotella) prevalence* |  | N1-methyladenosine | | 7813 | |  | | |  |
| *OTU99_155 (Butyrivibrio) abundance* |  | Inosine | | 2675 | |  | | |  |
| *OTU99_155 (Butyrivibrio) prevalence* |  | Hypoxanthine | | 7287 | |  | | |  |
| *OTU99_157 (Barnesiella) abundance* |  | Guanosine | | 2343 | |  | | |  |
| *OTU99_157 (Barnesiella) prevalence* |  | Allantoin | | 5705 | |  | | |  |
| *OTU99_16 (Faecalibacterium) abundance* |  | 7-methylguanine | | 6091 | |  | | |  |
| *OTU99_16 (Faecalibacterium) prevalence* |  | X-14450--phenylalanylleucine | | 2555 | |  | | |  |
| *OTU99_166 (Prevotella) abundance* |  | X-14304--leucylalanine | | 2434 | |  | | |  |
| *OTU99_166 (Prevotella) prevalence* |  | X-14208--phenylalanylserine | | 2455 | |  | | |  |
| *OTU99_17 (Parabacteroides) abundance* |  | X-14205--alpha-glutamyltyrosine | | 1789 | |  | | |  |
| *OTU99_17 (Parabacteroides) prevalence* |  | X-14189--leucylalanine | | 2745 | |  | | |  |
| *OTU99_171 (Bacteroides) abundance* |  | X-12244--N-acetylcarnosine | | 6608 | |  | | |  |
| *OTU99_171 (Bacteroides) prevalence* |  | Pyroglutamylglycine | | 1586 | |  | | |  |
| *OTU99_19 (Bacteria) abundance* |  | Pro-hydroxy-pro | | 7787 | |  | | |  |
| *OTU99_19 (Bacteria) prevalence* |  | Phenylalanylphenylalanine | | 4961 | |  | | |  |
| *OTU99_197 (Bacteroides) abundance* |  | Leucylleucine | | 3386 | |  | | |  |
| *OTU99_197 (Bacteroides) prevalence* |  | HWESASXX* | | 7700 | |  | | |  |
| *OTU99_2 (Bacteroides) abundance* |  | Glycylvaline | | 2108 | |  | | |  |
| *OTU99_2 (Bacteroides) prevalence* |  | Gamma-glutamylvaline | | 7753 | |  | | |  |
| *OTU99_21 (Parasutterella) abundance* |  | Gamma-glutamyltyrosine | | 7468 | |  | | |  |
| *OTU99_21 (Parasutterella) prevalence* |  | Gamma-glutamylthreonine* | | 4016 | |  | | |  |
| *OTU99_211 (Bacteroides) abundance* |  | Gamma-glutamylphenylalanine | | 7753 | |  | | |  |
| *OTU99_211 (Bacteroides) prevalence* |  | Gamma-glutamylmethionine* | | 2240 | |  | | |  |
| *OTU99_23 (Faecalibacterium) abundance* |  | Gamma-glutamylleucine | | 7802 | |  | | |  |
| *OTU99_23 (Faecalibacterium) prevalence* |  | Gamma-glutamylisoleucine* | | 5522 | |  | | |  |
| *OTU99_24 (Prevotella) abundance* |  | Gamma-glutamylglutamine | | 7662 | |  | | |  |
| *OTU99_24 (Prevotella) prevalence* |  | Gamma-glutamylglutamate | | 931 | |  | | |  |
| *OTU99_26 (Parasutterella) abundance* |  | DSGEGDFXAEGGGVR* | | 5371 | |  | | |  |
| *OTU99_26 (Parasutterella) prevalence* |  | Cyclo(leu-pro) | | 4833 | |  | | |  |
| *OTU99_271 (Prevotella) abundance* |  | Bradykinin, des-arg(9) | | 4570 | |  | | |  |
| *OTU99_271 (Prevotella) prevalence* |  | Aspartylphenylalanine | | 3948 | |  | | |  |
| *OTU99_298 (Phascolarctobacterium) abundance* |  | ADSGEGDFXAEGGGVR* | | 5588 | |  | | |  |
| *OTU99_298 (Phascolarctobacterium) prevalence* |  | ADpSGEGDFXAEGGGVR* | | 3939 | |  | | |  |
| *OTU99_3 (Bacteroides) abundance* |  | X-14977--vanillin | | 1789 | |  | | |  |
| *OTU99_3 (Bacteroides) prevalence* |  | Stachydrine | | 6745 | |  | | |  |
| *OTU99_30 (Parasutterella) abundance* |  | N-(2-furoyl)glycine | | 604 | |  | | |  |
| *OTU99_30 (Parasutterella) prevalence* |  | Homostachydrine* | | 3003 | |  | | |  |
| *OTU99_32 (Prevotella) abundance* |  | Ergothioneine | | 4453 | |  | | |  |
| *OTU99_32 (Prevotella) prevalence* |  | 4-hydroxyhippurate | | 4368 | |  | | |  |

**Table S2.** Mendelian randomization of gut microbiome on rheumatoid arthritis in European population (*P* < 1×10^-5^).

| **exposure** | **outcome** | **method** | **nsnp** | **b** | **se** | **pval** | **95%CI** | **p.adjust** |
| --- | --- | --- | --- | --- | --- | --- | --- | --- |
| **OTU99_5 (Sutterella) abundance** | **RA \|\| id: ukb-b-11874** | **IVW(M)** | **2** | **-6.89E-04** | **6.93E-05** | **2.74E-23** | **0.999 (0.999, 0.999)** | **1.12E-20** |
| **TestASV_6 (Subdoligranulum) abundance** | **RA \|\| id: ukb-b-11874** | **IVW(M)** | **2** | **-7.30E-04** | **9.91E-05** | **1.76E-13** | **0.999 (0.999, 0.999)** | **3.61E-11** |
| **G_Bacteroidetes prevalence** | **RA \|\| id: ukb-b-11874** | **IVW(M)** | **2** | **4.60E-04** | **7.40E-05** | **5.18E-10** | **1 (1, 1.001)** | **7.08E-08** |
| **OTU99_110 (Clostridiales) prevalence** | **RA \|\| id: ukb-b-11874** | **IVW(M)** | **2** | **9.99E-04** | **1.79E-04** | **2.43E-08** | **1.001 (1.001, 1.001)** | **2.49E-06** |
| **OTU99_6 (Sutterella) prevalence** | **RA \|\| id: ukb-b-11874** | **IVW(M)** | **2** | **1.13E-03** | **2.05E-04** | **3.45E-08** | **1.001 (1.001, 1.002)** | **2.83E-06** |
| **G_Prevotella abundance** | **RA \|\| id: ukb-b-11874** | **IVW(M)** | **6** | **-1.29E-03** | **2.71E-04** | **2.05E-06** | **0.999 (0.998, 0.999)** | **1.40E-04** |
| **OTU97_34 (Ruminococcus) abundance** | **RA \|\| id: ukb-b-11874** | **IVW(M)** | **3** | **-1.50E-03** | **3.48E-04** | **1.57E-05** | **0.998 (0.998, 0.999)** | **9.18E-04** |
| **TestASV_4 (Alistipes) prevalence** | **RA \|\| id: ukb-b-11874** | **IVW(M)** | **3** | **6.76E-04** | **1.59E-04** | **2.19E-05** | **1.001 (1, 1.001)** | **1.12E-03** |
| **G_Alloprevotella prevalence** | **RA \|\| id: ukb-b-11874** | **IVW(M)** | **9** | **-7.56E-04** | **1.95E-04** | **1.05E-04** | **0.999 (0.999, 1)** | **4.77E-03** |
| **OTU99_78 (Clostridiales) prevalence** | **RA \|\| id: ukb-b-11874** | **IVW(M)** | **4** | **-4.92E-04** | **1.40E-04** | **4.27E-04** | **1 (0.999, 1)** | **0.02** |
| **OTU97_69 (Clostridiales) prevalence** | **RA \|\| id: ukb-b-11874** | **IVW(M)** | **4** | **-4.93E-04** | **1.39E-04** | **4.12E-04** | **1 (0.999, 1)** | **0.02** |
| **TestASV_32 (Ruminococcaceae) prevalence** | **RA \|\| id: ukb-b-11874** | **IVW(M)** | **3** | **-2.45E-04** | **7.21E-05** | **6.71E-04** | **1 (1, 1)** | **0.02** |
| **G_Bacteroides abundance** | **RA \|\| id: ukb-b-11874** | **IVW(M)** | **7** | **-1.25E-03** | **3.98E-04** | **1.68E-03** | **0.999 (0.998, 1)** | **0.05** |
| **OTU99_110 (Clostridiales) abundance** | **RA \|\| id: ukb-b-11874** | **IVW(M)** | **6** | **1.56E-03** | **4.92E-04** | **1.56E-03** | **1.002 (1.001, 1.003)** | **0.05** |
| **F_Bacteroidaceae abundance** | **RA \|\| id: ukb-b-11874** | **IVW(M)** | **7** | **-1.25E-03** | **3.98E-04** | **1.68E-03** | **0.999 (0.998, 1)** | **0.05** |
| OTU99_65 (Sutterellaceae) prevalence | RA \|\| id: ukb-b-11874 | IVW(M) | 4 | 2.02E-04 | 6.81E-05 | 3.03E-03 | 1 (1, 1) | 0.06 |
| TestASV_47 (Parasutterella) prevalence | RA \|\| id: ukb-b-11874 | IVW(M) | 4 | 2.02E-04 | 6.81E-05 | 2.96E-03 | 1 (1, 1) | 0.06 |
| TestASV_43 (Parasutterella) abundance | RA \|\| id: ukb-b-11874 | IVW(M) | 7 | -4.44E-04 | 1.50E-04 | 3.01E-03 | 1 (0.999, 1) | 0.06 |
| OTU97_58 (Sutterellaceae) prevalence | RA \|\| id: ukb-b-11874 | IVW(M) | 4 | 2.02E-04 | 6.81E-05 | 3.03E-03 | 1 (1, 1) | 0.06 |
| OTU99_298 (Phascolarctobacterium) prevalence | RA \|\| id: ukb-b-11874 | IVW(M) | 5 | 2.77E-04 | 9.22E-05 | 2.69E-03 | 1 (1, 1) | 0.06 |
| TestASV_36 (Aestuariispira) prevalence | RA \|\| id: ukb-b-11874 | IVW(M) | 2 | 3.20E-04 | 1.07E-04 | 2.84E-03 | 1 (1, 1.001) | 0.06 |
| OTU97_235 (Phascolarctobacterium) prevalence | RA \|\| id: ukb-b-11874 | IVW(M) | 5 | 2.77E-04 | 9.22E-05 | 2.69E-03 | 1 (1, 1) | 0.06 |
| G_Ruminococcus2 abundance | RA \|\| id: ukb-b-11874 | IVW(M) | 2 | -1.24E-03 | 4.42E-04 | 4.94E-03 | 0.999 (0.998, 1) | 0.09 |
| OTU99_53 (Aestuariispira) prevalence | RA \|\| id: ukb-b-11874 | IVW(M) | 2 | 3.19E-04 | 1.15E-04 | 5.53E-03 | 1 (1, 1.001) | 0.09 |
| OTU97_50 (Aestuariispira) prevalence | RA \|\| id: ukb-b-11874 | IVW(M) | 2 | 3.19E-04 | 1.15E-04 | 5.53E-03 | 1 (1, 1.001) | 0.09 |
| OTU97_30 (Roseburia) abundance | RA \|\| id: ukb-b-11874 | IVW(M) | 6 | 8.48E-04 | 3.19E-04 | 7.86E-03 | 1.001 (1, 1.001) | 0.12 |
| OTU97_21 (Parasutterella) abundance | RA \|\| id: ukb-b-11874 | IVW(M) | 7 | -6.26E-04 | 2.37E-04 | 8.19E-03 | 0.999 (0.999, 1) | 0.12 |
| TestASV_42 (Alphaproteobacteria) prevalence | RA \|\| id: ukb-b-11874 | IVW(M) | 6 | -2.12E-04 | 8.46E-05 | 1.25E-02 | 1 (1, 1) | 0.17 |
| G_Parasutterella prevalence | RA \|\| id: ukb-b-11874 | IVW(M) | 3 | 6.17E-04 | 2.45E-04 | 1.17E-02 | 1.001 (1, 1.001) | 0.17 |
| OTU97_65 (Parabacteroides) abundance | RA \|\| id: ukb-b-11874 | IVW(M) | 4 | -7.20E-04 | 2.88E-04 | 1.25E-02 | 0.999 (0.999, 1) | 0.17 |
| OTU97_15 (Parasutterella) abundance | RA \|\| id: ukb-b-11874 | IVW(M) | 9 | 6.20E-04 | 2.53E-04 | 1.41E-02 | 1.001 (1, 1.001) | 0.19 |
| G_Bacteroidales prevalence | RA \|\| id: ukb-b-11874 | IVW(M) | 5 | -4.41E-04 | 1.82E-04 | 1.55E-02 | 1 (0.999, 1) | 0.19 |
| OTU99_120 (Ruminococcaceae) prevalence | RA \|\| id: ukb-b-11874 | IVW(M) | 3 | 3.77E-04 | 1.55E-04 | 1.53E-02 | 1 (1, 1.001) | 0.19 |
| OTU99_110 (Clostridiales) abundance | RA \|\| id: ukb-b-11874 | IVW (F) | 6 | 1.56E-03 | 4.71E-04 | 9.61E-04 | 1.002 (1.001, 1.002) | 0.20 |
| G_Alloprevotella prevalence | RA \|\| id: ukb-b-11874 | IVW (F) | 9 | -7.56E-04 | 2.26E-04 | 8.02E-04 | 0.999 (0.999, 1) | 0.20 |
| OTU99_62 (Ruminococcaceae) prevalence | RA \|\| id: ukb-b-11874 | IVW(M) | 4 | 7.07E-04 | 2.98E-04 | 1.78E-02 | 1.001 (1, 1.001) | 0.21 |
| TestASV_11 (Lachnospiraceae) abundance | RA \|\| id: ukb-b-11874 | IVW(M) | 5 | 8.37E-04 | 3.55E-04 | 1.82E-02 | 1.001 (1, 1.002) | 0.21 |
| TestASV_14 (Lachnospiraceae) prevalence | RA \|\| id: ukb-b-11874 | IVW(M) | 4 | 3.28E-04 | 1.44E-04 | 2.23E-02 | 1 (1, 1.001) | 0.25 |
| TestASV_35 (Alphaproteobacteria) abundance | RA \|\| id: ukb-b-11874 | IVW(M) | 3 | 6.30E-05 | 2.77E-05 | 2.31E-02 | 1 (1, 1) | 0.26 |
| TestASV_13 (Bacteroides) abundance | RA \|\| id: ukb-b-11874 | IVW(M) | 7 | 7.91E-04 | 3.50E-04 | 2.40E-02 | 1.001 (1, 1.001) | 0.26 |
| OTU97_16 (Faecalibacterium) prevalence | RA \|\| id: ukb-b-11874 | IVW(M) | 5 | -4.80E-04 | 2.14E-04 | 2.48E-02 | 1 (0.999, 1) | 0.26 |
| OTU99_98 (Phascolarctobacterium) prevalence | RA \|\| id: ukb-b-11874 | IVW(M) | 3 | 5.20E-04 | 2.33E-04 | 2.59E-02 | 1.001 (1, 1.001) | 0.27 |
| OTU99_110 (Clostridiales) abundance | RA \|\| id: ukb-b-11874 | Maximum likelihood | 6 | 1.62E-03 | 5.06E-04 | 1.36E-03 | 1.002 (1.001, 1.003) | 0.27 |
| G_Alloprevotella prevalence | RA \|\| id: ukb-b-11874 | Maximum likelihood | 9 | -7.73E-04 | 2.37E-04 | 1.09E-03 | 0.999 (0.999, 1) | 0.27 |
| OTU99_19 (Bacteria) abundance | RA \|\| id: ukb-b-11874 | IVW(M) | 6 | -4.35E-04 | 1.97E-04 | 2.70E-02 | 1 (0.999, 1) | 0.27 |
| OTU99_8 (Ruminococcaceae) prevalence | RA \|\| id: ukb-b-11874 | IVW(M) | 3 | 5.48E-04 | 2.51E-04 | 2.93E-02 | 1.001 (1, 1.001) | 0.28 |
| OTU97_163 (Bacteroides) prevalence | RA \|\| id: ukb-b-11874 | IVW(M) | 8 | -2.50E-04 | 1.15E-04 | 2.97E-02 | 1 (1, 1) | 0.28 |
| OTU99_85 (Alistipes) abundance | RA \|\| id: ukb-b-11874 | IVW(M) | 12 | 2.68E-04 | 1.24E-04 | 3.12E-02 | 1 (1, 1.001) | 0.29 |
| TestASV_16 (Bacteroides) abundance | RA \|\| id: ukb-b-11874 | IVW(M) | 4 | -7.81E-04 | 3.68E-04 | 3.38E-02 | 0.999 (0.998, 1) | 0.31 |
| TestASV_18 (Prevotella) prevalence | RA \|\| id: ukb-b-11874 | IVW(M) | 3 | 5.09E-04 | 2.44E-04 | 3.66E-02 | 1.001 (1, 1.001) | 0.32 |
| OTU99_39 (Alphaproteobacteria) abundance | RA \|\| id: ukb-b-11874 | IVW(M) | 3 | -1.05E-03 | 5.03E-04 | 3.69E-02 | 0.999 (0.998, 1) | 0.32 |
| OTU99_211 (Bacteroides) abundance | RA \|\| id: ukb-b-11874 | IVW(M) | 4 | -3.77E-04 | 1.80E-04 | 3.67E-02 | 1 (0.999, 1) | 0.32 |
| G_Porphyromonadaceae abundance | RA \|\| id: ukb-b-11874 | IVW(M) | 9 | -7.92E-04 | 3.82E-04 | 3.80E-02 | 0.999 (0.998, 1) | 0.32 |
| OTU99_110 (Clostridiales) abundance | RA \|\| id: ukb-b-11874 | IVW | 6 | 1.56E-03 | 4.92E-04 | 1.56E-03 | 1.002 (1.001, 1.003) | 0.32 |
| G_Alloprevotella prevalence | RA \|\| id: ukb-b-11874 | IVW | 9 | -7.56E-04 | 2.26E-04 | 8.02E-04 | 0.999 (0.999, 1) | 0.32 |
| OTU99_116 (Sutterella) abundance | RA \|\| id: ukb-b-11874 | IVW(M) | 2 | 5.53E-04 | 2.69E-04 | 4.01E-02 | 1.001 (1, 1.001) | 0.33 |
| TestASV_27 (Ruminococcaceae) prevalence | RA \|\| id: ukb-b-11874 | IVW(M) | 4 | -5.35E-04 | 2.66E-04 | 4.40E-02 | 0.999 (0.999, 1) | 0.35 |
| G_Parabacteroides prevalence | RA \|\| id: ukb-b-11874 | IVW(M) | 5 | -2.22E-04 | 1.10E-04 | 4.45E-02 | 1 (1, 1) | 0.35 |
| OTU99_171 (Bacteroides) abundance | RA \|\| id: ukb-b-11874 | IVW(M) | 6 | 1.36E-03 | 6.87E-04 | 4.77E-02 | 1.001 (1, 1.003) | 0.37 |
| TestASV_30 (Paraprevotella) abundance | RA \|\| id: ukb-b-11874 | IVW(M) | 8 | 1.67E-04 | 8.48E-05 | 4.94E-02 | 1 (1, 1) | 0.37 |

RA, rheumatoid arthritis; IVW, Inverse variance weighted; IVW(M), Inverse variance weighted (multiplicative random effects); IVW (F), Inverse variance weighted (F); nsnp is the number of SNPs being used as IVs; 95%CI: confidence interval; pval of the intercept from the method; p.adjust: the p value after using the Benjamini-Hochberg method; significant p.adjust were marked in bold.

**Table S3.** Mendelian randomization of rheumatoid arthritis on microbial traits in European population.

| **outcome** | **exposure** | **method** | **nsnp** | **b** | **se** | **pval** | **p.adjust** |
| --- | --- | --- | --- | --- | --- | --- | --- |
| **OTU99_45 (Faecalibacterium) prevalence** | **RA \|\| id:ukb-b-11874** | **IVW(M)** | **2** | **24.60** | **0.09** | **0.00E+00** | **0.00E+00** |
| **OTU99_84 (Prevotella) prevalence** | **RA \|\| id:ukb-b-11874** | **IVW(M)** | **2** | **25.95** | **0.18** | **0.00E+00** | **0.00E+00** |
| **OTU99_271 (Prevotella) prevalence** | **RA \|\| id:ukb-b-11874** | **IVW(M)** | **2** | **-34.09** | **0.10** | **0.00E+00** | **0.00E+00** |
| **TestASV_22 (Sutterella) prevalence** | **RA \|\| id:ukb-b-11874** | **IVW(M)** | **2** | **8.42** | **0.20** | **0.00E+00** | **0.00E+00** |
| **TestASV_9 (Alistipes) abundance** | **RA \|\| id:ukb-b-11874** | **IVW(M)** | **2** | **21.16** | **0.48** | **0.00E+00** | **0.00E+00** |
| **OTU99_92 (Ruminococcus) abundance** | **RA \|\| id:ukb-b-11874** | **IVW(M)** | **2** | **16.89** | **0.54** | **2.73E-212** | **1.95E-210** |
| **G_Barnesiella prevalence** | **RA \|\| id:ukb-b-11874** | **IVW(M)** | **2** | **-17.72** | **0.58** | **1.50E-207** | **9.20E-206** |
| **OTU99_108 (Cytophagales) prevalence** | **RA \|\| id:ukb-b-11874** | **IVW(M)** | **2** | **36.38** | **1.46** | **4.33E-137** | **2.32E-135** |
| **OTU97_95 (Cytophagales) abundance** | **RA \|\| id:ukb-b-11874** | **IVW(M)** | **2** | **17.67** | **0.77** | **1.65E-117** | **7.87E-116** |
| **OTU97_354 (Alloprevotella) prevalence** | **RA \|\| id:ukb-b-11874** | **IVW(M)** | **2** | **-105.64** | **4.83** | **5.81E-106** | **2.49E-104** |
| **TestASV_13 (Bacteroides) prevalence** | **RA \|\| id:ukb-b-11874** | **IVW(M)** | **2** | **-42.16** | **2.00** | **4.27E-99** | **1.67E-97** |
| **OTU97_109 (Paraprevotella) prevalence** | **RA \|\| id:ukb-b-11874** | **IVW(M)** | **2** | **-9.40** | **0.56** | **4.29E-63** | **1.53E-61** |
| **OTU99_47 (Prevotella) prevalence** | **RA \|\| id:ukb-b-11874** | **IVW(M)** | **2** | **31.65** | **1.90** | **4.64E-62** | **1.53E-60** |
| **OTU99_150 (Prevotella) abundance** | **RA \|\| id:ukb-b-11874** | **IVW(M)** | **2** | **9.88** | **0.62** | **3.17E-57** | **9.71E-56** |
| **OTU97_95 (Cytophagales) prevalence** | **RA \|\| id:ukb-b-11874** | **IVW(M)** | **2** | **34.20** | **2.31** | **1.09E-49** | **3.11E-48** |
| **OTU97_65 (Parabacteroides) prevalence** | **RA \|\| id:ukb-b-11874** | **IVW(M)** | **2** | **17.59** | **1.23** | **4.03E-46** | **1.08E-44** |
| **OTU97_12 (Bacteroides) prevalence** | **RA \|\| id:ukb-b-11874** | **IVW(M)** | **2** | **-41.72** | **3.00** | **6.10E-44** | **1.45E-42** |
| **OTU99_12 (Bacteroides) prevalence** | **RA \|\| id:ukb-b-11874** | **IVW(M)** | **2** | **-41.72** | **3.00** | **6.10E-44** | **1.45E-42** |
| **OTU99_85 (Alistipes) prevalence** | **RA \|\| id:ukb-b-11874** | **IVW(M)** | **2** | **16.86** | **1.28** | **2.09E-39** | **4.73E-38** |
| **OTU99_17 (Parabacteroides) prevalence** | **RA \|\| id:ukb-b-11874** | **IVW(M)** | **2** | **41.92** | **3.37** | **1.38E-35** | **2.95E-34** |
| **F_Lachnospiraceae abundance** | **RA \|\| id:ukb-b-11874** | **IVW(M)** | **2** | **3.54** | **0.31** | **1.75E-30** | **3.58E-29** |
| **OTU97_140 (Bacteroides) abundance** | **RA \|\| id:ukb-b-11874** | **IVW(M)** | **2** | **-19.59** | **1.75** | **5.45E-29** | **1.06E-27** |
| **OTU99_108 (Cytophagales) abundance** | **RA \|\| id:ukb-b-11874** | **IVW(M)** | **2** | **3.79** | **0.36** | **1.97E-26** | **3.67E-25** |
| **TestASV_18 (Prevotella) abundance** | **RA \|\| id:ukb-b-11874** | **IVW(M)** | **2** | **10.00** | **0.95** | **6.60E-26** | **1.18E-24** |
| **TestASV_11 (Lachnospiraceae) abundance** | **RA \|\| id:ukb-b-11874** | **IVW(M)** | **2** | **20.88** | **2.02** | **4.33E-25** | **7.43E-24** |
| **C_Alphaproteobacteria prevalence** | **RA \|\| id:ukb-b-11874** | **IVW(M)** | **2** | **-14.26** | **1.42** | **1.12E-23** | **1.85E-22** |
| **F_Bacteroidaceae abundance** | **RA \|\| id:ukb-b-11874** | **IVW(M)** | **2** | **-5.55** | **0.56** | **2.97E-23** | **4.59E-22** |
| **G_Bacteroides abundance** | **RA \|\| id:ukb-b-11874** | **IVW(M)** | **2** | **-5.55** | **0.56** | **3.00E-23** | **4.59E-22** |
| **OTU99_35 (Ruminococcus) prevalence** | **RA \|\| id:ukb-b-11874** | **IVW(M)** | **2** | **30.62** | **3.13** | **1.22E-22** | **1.81E-21** |
| **OTU97_51 (Barnesiella) prevalence** | **RA \|\| id:ukb-b-11874** | **IVW(M)** | **2** | **-19.23** | **2.08** | **2.38E-20** | **3.40E-19** |
| **TestASV_1 (Bacteroides) abundance** | **RA \|\| id:ukb-b-11874** | **IVW(M)** | **2** | **10.72** | **1.18** | **1.27E-19** | **1.75E-18** |
| **G_Subdoligranulum abundance** | **RA \|\| id:ukb-b-11874** | **IVW(M)** | **2** | **-3.24** | **0.40** | **4.85E-16** | **6.50E-15** |
| **OTU97_119 (Desulfovibrio) abundance** | **RA \|\| id:ukb-b-11874** | **IVW(M)** | **2** | **10.18** | **1.26** | **6.60E-16** | **8.58E-15** |
| **OTU97_74 (Alistipes) abundance** | **RA \|\| id:ukb-b-11874** | **IVW(M)** | **2** | **14.14** | **1.90** | **8.91E-14** | **1.12E-12** |
| **OTU99_106 (Barnesiella) prevalence** | **RA \|\| id:ukb-b-11874** | **IVW(M)** | **2** | **-31.48** | **4.54** | **3.91E-12** | **4.79E-11** |
| **OTU97_34 (Ruminococcus) prevalence** | **RA \|\| id:ukb-b-11874** | **IVW(M)** | **2** | **32.99** | **4.76** | **4.15E-12** | **4.94E-11** |
| **OTU97_11 (Parabacteroides) abundance** | **RA \|\| id:ukb-b-11874** | **IVW(M)** | **2** | **15.54** | **2.26** | **6.46E-12** | **7.49E-11** |
| **OTU99_110 (Clostridiales) abundance** | **RA \|\| id:ukb-b-11874** | **IVW(M)** | **2** | **15.33** | **2.24** | **8.47E-12** | **9.56E-11** |
| **TestASV_26 (Phascolarctobacterium) prevalence** | **RA \|\| id:ukb-b-11874** | **IVW(M)** | **2** | **-72.94** | **10.93** | **2.46E-11** | **2.70E-10** |
| **OTU99_124 (Paraprevotella) prevalence** | **RA \|\| id:ukb-b-11874** | **IVW(M)** | **2** | **-12.87** | **1.96** | **4.94E-11** | **5.29E-10** |
| **OTU99_19 (Bacteria) abundance** | **RA \|\| id:ukb-b-11874** | **IVW(M)** | **2** | **-16.96** | **2.61** | **7.71E-11** | **8.07E-10** |
| **TestASV_39 (Bacteroides) prevalence** | **RA \|\| id:ukb-b-11874** | **IVW(M)** | **2** | **-76.61** | **12.76** | **1.94E-09** | **1.98E-08** |
| **G_Holdemanella abundance** | **RA \|\| id:ukb-b-11874** | **IVW(M)** | **2** | **-17.56** | **3.34** | **1.49E-07** | **1.48E-06** |
| **C_Negativicutes abundance** | **RA \|\| id:ukb-b-11874** | **IVW(M)** | **2** | **-14.88** | **2.84** | **1.57E-07** | **1.50E-06** |
| **O_Selenomonadales abundance** | **RA \|\| id:ukb-b-11874** | **IVW(M)** | **2** | **-14.88** | **2.84** | **1.57E-07** | **1.50E-06** |
| **G_Parasutterella prevalence** | **RA \|\| id:ukb-b-11874** | **IVW(M)** | **2** | **27.08** | **5.18** | **1.76E-07** | **1.64E-06** |
| **OTU99_558 (Bacteroidales) prevalence** | **RA \|\| id:ukb-b-11874** | **IVW(M)** | **2** | **-33.19** | **6.38** | **1.98E-07** | **1.81E-06** |
| **OTU99_13 (Subdoligranulum) prevalence** | **RA \|\| id:ukb-b-11874** | **IVW(M)** | **2** | **27.56** | **5.59** | **8.16E-07** | **7.29E-06** |
| **TestASV_14 (Lachnospiraceae) abundance** | **RA \|\| id:ukb-b-11874** | **IVW(M)** | **2** | **17.18** | **3.61** | **1.94E-06** | **1.70E-05** |
| **G_Prevotella abundance** | **RA \|\| id:ukb-b-11874** | **IVW(M)** | **2** | **10.94** | **2.31** | **2.20E-06** | **1.89E-05** |
| **OTU97_56 (Ruminococcaceae) prevalence** | **RA \|\| id:ukb-b-11874** | **IVW(M)** | **2** | **-28.37** | **6.00** | **2.30E-06** | **1.94E-05** |
| **OTU97_33 (Holdemanella) abundance** | **RA \|\| id:ukb-b-11874** | **IVW(M)** | **2** | **-19.25** | **4.25** | **6.03E-06** | **4.97E-05** |
| **P_Firmicutes abundance** | **RA \|\| id:ukb-b-11874** | **IVW(M)** | **2** | **-5.89** | **1.31** | **6.58E-06** | **5.33E-05** |
| **G_Ruminococcaceae abundance** | **RA \|\| id:ukb-b-11874** | **IVW(M)** | **2** | **14.78** | **3.34** | **9.53E-06** | **7.57E-05** |
| **OTU99_121 (Ruminococcaceae) prevalence** | **RA \|\| id:ukb-b-11874** | **IVW(M)** | **2** | **43.54** | **10.18** | **1.89E-05** | **1.48E-04** |
| **OTU99_72 (Parabacteroides) prevalence** | **RA \|\| id:ukb-b-11874** | **IVW(M)** | **2** | **16.54** | **3.88** | **2.00E-05** | **1.53E-04** |
| **OTU97_15 (Parasutterella) prevalence** | **RA \|\| id:ukb-b-11874** | **IVW(M)** | **2** | **12.88** | **3.04** | **2.24E-05** | **1.69E-04** |
| **OTU97_106 (Ruminococcaceae) prevalence** | **RA \|\| id:ukb-b-11874** | **IVW(M)** | **2** | **44.34** | **10.53** | **2.55E-05** | **1.89E-04** |
| **OTU99_55 (Barnesiella) prevalence** | **RA \|\| id:ukb-b-11874** | **IVW(M)** | **2** | **17.88** | **4.64** | **1.15E-04** | **8.37E-04** |
| **OTU97_102 (Prevotella) abundance** | **RA \|\| id:ukb-b-11874** | **IVW(M)** | **2** | **-18.45** | **4.88** | **1.55E-04** | **1.11E-03** |
| **OTU97_80 (Ruminococcus) abundance** | **RA \|\| id:ukb-b-11874** | **IVW(M)** | **2** | **13.52** | **3.75** | **3.07E-04** | **2.16E-03** |
| **F_Prevotellaceae abundance** | **RA \|\| id:ukb-b-11874** | **IVW(M)** | **2** | **-5.63** | **1.58** | **3.55E-04** | **2.42E-03** |
| **OTU97_102 (Prevotella) prevalence** | **RA \|\| id:ukb-b-11874** | **IVW(M)** | **2** | **-6.96** | **1.95** | **3.55E-04** | **2.42E-03** |
| **OTU97_30 (Roseburia) prevalence** | **RA \|\| id:ukb-b-11874** | **IVW(M)** | **2** | **-29.01** | **8.20** | **4.00E-04** | **2.68E-03** |
| **G_Prevotellaceae abundance** | **RA \|\| id:ukb-b-11874** | **IVW(M)** | **2** | **14.40** | **4.28** | **7.70E-04** | **0.01** |
| **OTU99_62 (Ruminococcaceae) prevalence** | **RA \|\| id:ukb-b-11874** | **IVW(M)** | **2** | **-25.80** | **7.72** | **8.33E-04** | **0.01** |
| **C_Bacteroidia abundance** | **RA \|\| id:ukb-b-11874** | **IVW(M)** | **2** | **-5.92** | **1.78** | **8.58E-04** | **0.01** |
| **O_Bacteroidales abundance** | **RA \|\| id:ukb-b-11874** | **IVW(M)** | **2** | **-5.92** | **1.78** | **8.58E-04** | **0.01** |
| **OTU97_277 (Prevotella) prevalence** | **RA \|\| id:ukb-b-11874** | **IVW(M)** | **2** | **-9.19** | **2.76** | **8.88E-04** | **0.01** |
| **G_Paraprevotella abundance** | **RA \|\| id:ukb-b-11874** | **IVW(M)** | **2** | **-4.90** | **1.50** | **1.10E-03** | **0.01** |
| **OTU99_55 (Barnesiella) abundance** | **RA \|\| id:ukb-b-11874** | **IVW(M)** | **2** | **-22.84** | **7.19** | **1.48E-03** | **0.01** |
| **G_Alphaproteobacteria prevalence** | **RA \|\| id:ukb-b-11874** | **IVW(M)** | **2** | **-14.92** | **4.77** | **1.77E-03** | **0.01** |
| **OTU99_21 (Parasutterella) prevalence** | **RA \|\| id:ukb-b-11874** | **IVW(M)** | **2** | **13.38** | **4.31** | **1.90E-03** | **0.01** |
| **OTU99_23 (Faecalibacterium) prevalence** | **RA \|\| id:ukb-b-11874** | **IVW(M)** | **2** | **18.70** | **6.05** | **1.99E-03** | **0.01** |
| **OTU99_120 (Ruminococcaceae) prevalence** | **RA \|\| id:ukb-b-11874** | **IVW(M)** | **2** | **29.04** | **9.53** | **2.30E-03** | **0.01** |
| **OTU99_6 (Sutterella) prevalence** | **RA \|\| id:ukb-b-11874** | **IVW(M)** | **2** | **8.43** | **2.77** | **2.32E-03** | **0.01** |
| **G_Sutterella abundance** | **RA \|\| id:ukb-b-11874** | **IVW(M)** | **2** | **-11.85** | **4.23** | **0.01** | **0.03** |
| **G_Ruminococcus2 abundance** | **RA \|\| id:ukb-b-11874** | **IVW(M)** | **2** | **5.99** | **2.15** | **0.01** | **0.03** |
| **OTU99_132 (Bacteroidales) abundance** | **RA \|\| id:ukb-b-11874** | **IVW(M)** | **2** | **5.12** | **1.85** | **0.01** | **0.03** |
| OTU97_354 (Alloprevotella) prevalence | RA \|\| id:ukb-b-11874 | IVW(F) | 2 | -105.64 | 38.68 | 0.01 | 0.97 |
| OTU97_354 (Alloprevotella) prevalence | RA \|\| id:ukb-b-11874 | IVW | 2 | -105.64 | 38.68 | 0.01 | 0.99 |
| OTU97_354 (Alloprevotella) prevalence | RA \|\| id:ukb-b-11874 | ML | 2 | -105.63 | 39.12 | 0.01 | 0.97 |
| **OTU97_97 (Clostridiales) prevalence** | **RA \|\| id:ukb-b-11874** | **IVW(M)** | **2** | **-4.00** | **1.51** | **0.01** | **0.04** |
| **OTU97_46 (Lachnospiraceae) prevalence** | **RA \|\| id:ukb-b-11874** | **IVW(M)** | **2** | **-11.83** | **4.46** | **0.01** | **0.04** |
| **TestASV_1 (Bacteroides) prevalence** | **RA \|\| id:ukb-b-11874** | **IVW(M)** | **2** | **19.72** | **7.54** | **0.01** | **0.05** |
| **G_Sutterella prevalence** | **RA \|\| id:ukb-b-11874** | **IVW(M)** | **2** | **-15.76** | **6.06** | **0.01** | **0.05** |
| TestASV_23 (Barnesiella) prevalence | RA \|\| id:ukb-b-11874 | IVW(M) | 2 | 4.53 | 1.76 | 0.01 | 0.05 |
| OTU99_171 (Bacteroides) abundance | RA \|\| id:ukb-b-11874 | IVW(M) | 2 | -15.45 | 6.11 | 0.01 | 0.06 |
| TestASV_21 (Ruminococcaceae) prevalence | RA \|\| id:ukb-b-11874 | IVW(M) | 2 | 14.07 | 5.58 | 0.01 | 0.06 |
| OTU97_106 (Ruminococcaceae) prevalence | RA \|\| id:ukb-b-11874 | IVW(F) | 2 | 44.34 | 17.60 | 0.01 | 0.97 |
| OTU97_106 (Ruminococcaceae) prevalence | RA \|\| id:ukb-b-11874 | IVW | 2 | 44.34 | 17.60 | 0.01 | 0.99 |
| OTU97_58 (Sutterellaceae) abundance | RA \|\| id:ukb-b-11874 | IVW(M) | 2 | -17.46 | 7.00 | 0.01 | 0.06 |
| OTU97_106 (Ruminococcaceae) prevalence | RA \|\| id:ukb-b-11874 | ML | 2 | 44.39 | 17.80 | 0.01 | 0.97 |
| OTU99_65 (Sutterellaceae) abundance | RA \|\| id:ukb-b-11874 | IVW(M) | 2 | -17.33 | 6.98 | 0.01 | 0.06 |
| OTU99_121 (Ruminococcaceae) prevalence | RA \|\| id:ukb-b-11874 | IVW(F) | 2 | 43.54 | 17.62 | 0.01 | 0.97 |
| OTU99_121 (Ruminococcaceae) prevalence | RA \|\| id:ukb-b-11874 | IVW | 2 | 43.54 | 17.62 | 0.01 | 0.99 |
| TestASV_14 (Lachnospiraceae) prevalence | RA \|\| id:ukb-b-11874 | IVW(M) | 2 | -23.75 | 9.67 | 0.01 | 0.07 |
| OTU99_121 (Ruminococcaceae) prevalence | RA \|\| id:ukb-b-11874 | ML | 2 | 43.59 | 17.81 | 0.01 | 0.97 |
| OTU99_124 (Paraprevotella) abundance | RA \|\| id:ukb-b-11874 | IVW(M) | 2 | 28.88 | 11.82 | 0.01 | 0.07 |
| O_Coriobacteriales abundance | RA \|\| id:ukb-b-11874 | IVW(M) | 2 | 5.49 | 2.28 | 0.02 | 0.07 |
| F_Coriobacteriaceae abundance | RA \|\| id:ukb-b-11874 | IVW(M) | 2 | 5.49 | 2.28 | 0.02 | 0.07 |
| OTU99_78 (Clostridiales) prevalence | RA \|\| id:ukb-b-11874 | IVW(M) | 2 | -23.79 | 9.94 | 0.02 | 0.08 |
| OTU99_84 (Prevotella) abundance | RA \|\| id:ukb-b-11874 | IVW(M) | 2 | 12.55 | 5.25 | 0.02 | 0.08 |
| OTU97_6 (Sutterella) prevalence | RA \|\| id:ukb-b-11874 | IVW(M) | 2 | 11.80 | 4.94 | 0.02 | 0.08 |
| TestASV_46 (Sutterella) prevalence | RA \|\| id:ukb-b-11874 | IVW(M) | 2 | -69.07 | 29.13 | 0.02 | 0.08 |
| OTU97_13 (Subdoligranulum) prevalence | RA \|\| id:ukb-b-11874 | IVW(M) | 2 | 27.35 | 12.00 | 0.02 | 0.10 |
| OTU97_3 (Bacteroides) prevalence | RA \|\| id:ukb-b-11874 | IVW(M) | 2 | -44.20 | 19.61 | 0.02 | 0.10 |
| OTU99_3 (Bacteroides) prevalence | RA \|\| id:ukb-b-11874 | IVW(M) | 2 | -44.20 | 19.61 | 0.02 | 0.10 |
| OTU97_105 (Ruminococcaceae) abundance | RA \|\| id:ukb-b-11874 | IVW(M) | 2 | 11.38 | 5.09 | 0.03 | 0.11 |
| OTU97_69 (Clostridiales) prevalence | RA \|\| id:ukb-b-11874 | IVW(M) | 2 | -22.32 | 10.07 | 0.03 | 0.11 |
| OTU99_120 (Ruminococcaceae) abundance | RA \|\| id:ukb-b-11874 | IVW(M) | 2 | 11.58 | 5.26 | 0.03 | 0.12 |
| OTU97_12 (Bacteroides) abundance | RA \|\| id:ukb-b-11874 | IVW(M) | 2 | 11.58 | 5.26 | 0.03 | 0.12 |
| OTU97_108 (Phascolarctobacterium) abundance | RA \|\| id:ukb-b-11874 | IVW(M) | 2 | 27.60 | 12.56 | 0.03 | 0.12 |
| OTU99_47 (Prevotella) abundance | RA \|\| id:ukb-b-11874 | IVW(M) | 2 | -6.72 | 3.06 | 0.03 | 0.12 |
| OTU99_1 (Bacteroides) prevalence | RA \|\| id:ukb-b-11874 | IVW(M) | 2 | 42.39 | 19.38 | 0.03 | 0.12 |
| TestASV_24 (Bacteroides) prevalence | RA \|\| id:ukb-b-11874 | IVW(F) | 2 | 64.97 | 29.77 | 0.03 | 0.97 |
| TestASV_24 (Bacteroides) prevalence | RA \|\| id:ukb-b-11874 | ML | 2 | 65.15 | 29.99 | 0.03 | 0.97 |
| OTU99_17 (Parabacteroides) prevalence | RA \|\| id:ukb-b-11874 | IVW(F) | 2 | 41.92 | 19.38 | 0.03 | 0.97 |
| OTU99_17 (Parabacteroides) prevalence | RA \|\| id:ukb-b-11874 | IVW | 2 | 41.92 | 19.38 | 0.03 | 0.99 |
| G_Bacteroidetes abundance | RA \|\| id:ukb-b-11874 | IVW(M) | 2 | 11.86 | 5.49 | 0.03 | 0.12 |
| OTU99_17 (Parabacteroides) prevalence | RA \|\| id:ukb-b-11874 | ML | 2 | 41.93 | 19.52 | 0.03 | 0.97 |
| TestASV_13 (Bacteroides) prevalence | RA \|\| id:ukb-b-11874 | IVW(F) | 2 | -42.16 | 19.77 | 0.03 | 0.97 |
| TestASV_13 (Bacteroides) prevalence | RA \|\| id:ukb-b-11874 | IVW | 2 | -42.16 | 19.77 | 0.03 | 0.99 |
| OTU97_1 (Bacteroides) prevalence | RA \|\| id:ukb-b-11874 | IVW(M) | 2 | 42.32 | 19.92 | 0.03 | 0.13 |
| TestASV_13 (Bacteroides) prevalence | RA \|\| id:ukb-b-11874 | ML | 2 | -42.16 | 19.91 | 0.03 | 0.97 |
| OTU97_12 (Bacteroides) prevalence | RA \|\| id:ukb-b-11874 | IVW(F) | 2 | -41.72 | 19.72 | 0.03 | 0.97 |
| OTU99_12 (Bacteroides) prevalence | RA \|\| id:ukb-b-11874 | IVW(F) | 2 | -41.72 | 19.72 | 0.03 | 0.97 |
| OTU97_12 (Bacteroides) prevalence | RA \|\| id:ukb-b-11874 | IVW | 2 | -41.72 | 19.72 | 0.03 | 0.99 |
| OTU99_12 (Bacteroides) prevalence | RA \|\| id:ukb-b-11874 | IVW | 2 | -41.72 | 19.72 | 0.03 | 0.99 |
| TestASV_27 (Ruminococcaceae) abundance | RA \|\| id:ukb-b-11874 | ML | 2 | 39.97 | 18.96 | 0.03 | 0.97 |
| TestASV_27 (Ruminococcaceae) abundance | RA \|\| id:ukb-b-11874 | IVW(F) | 2 | 39.49 | 18.74 | 0.04 | 0.97 |
| TestASV_37 (Ruminococcaceae) prevalence | RA \|\| id:ukb-b-11874 | IVW(M) | 2 | -41.91 | 19.91 | 0.04 | 0.14 |
| OTU97_12 (Bacteroides) prevalence | RA \|\| id:ukb-b-11874 | ML | 2 | -41.72 | 19.86 | 0.04 | 0.97 |
| OTU99_12 (Bacteroides) prevalence | RA \|\| id:ukb-b-11874 | ML | 2 | -41.72 | 19.86 | 0.04 | 0.97 |
| OTU97_101 (Sutterella) prevalence | RA \|\| id:ukb-b-11874 | IVW(M) | 2 | -59.21 | 28.19 | 0.04 | 0.14 |
| OTU99_116 (Sutterella) prevalence | RA \|\| id:ukb-b-11874 | IVW(M) | 2 | -59.21 | 28.19 | 0.04 | 0.14 |
| TestASV_39 (Bacteroides) prevalence | RA \|\| id:ukb-b-11874 | IVW(F) | 2 | -76.61 | 36.52 | 0.04 | 0.97 |
| TestASV_39 (Bacteroides) prevalence | RA \|\| id:ukb-b-11874 | IVW | 2 | -76.61 | 36.52 | 0.04 | 0.99 |
| TestASV_39 (Bacteroides) prevalence | RA \|\| id:ukb-b-11874 | ML | 2 | -76.63 | 36.78 | 0.04 | 0.97 |
| OTU99_39 (Alphaproteobacteria) abundance | RA \|\| id:ukb-b-11874 | IVW(M) | 2 | 18.50 | 8.99 | 0.04 | 0.15 |
| OTU97_3 (Bacteroides) abundance | RA \|\| id:ukb-b-11874 | IVW(M) | 2 | 7.21 | 3.55 | 0.04 | 0.16 |
| TestASV_26 (Phascolarctobacterium) prevalence | RA \|\| id:ukb-b-11874 | IVW(F) | 2 | -72.94 | 35.87 | 0.04 | 0.97 |
| TestASV_26 (Phascolarctobacterium) prevalence | RA \|\| id:ukb-b-11874 | IVW | 2 | -72.94 | 35.87 | 0.04 | 0.99 |
| G_Lachnospiraceae abundance | RA \|\| id:ukb-b-11874 | IVW(M) | 2 | -2.61 | 1.28 | 0.04 | 0.16 |
| TestASV_26 (Phascolarctobacterium) prevalence | RA \|\| id:ukb-b-11874 | ML | 2 | -72.97 | 36.11 | 0.04 | 0.97 |
| TestASV_45 (Clostridiales) prevalence | RA \|\| id:ukb-b-11874 | IVW(M) | 2 | -34.61 | 17.17 | 0.04 | 0.16 |
| OTU97_24 (Prevotella) prevalence | RA \|\| id:ukb-b-11874 | IVW(F) | 2 | 49.88 | 25.13 | 0.05 | 0.97 |
| OTU97_17 (Parabacteroides) prevalence | RA \|\| id:ukb-b-11874 | IVW(M) | 2 | 20.22 | 10.19 | 0.05 | 0.17 |
| OTU99_30 (Parasutterella) abundance | RA \|\| id:ukb-b-11874 | IVW(M) | 2 | 6.87 | 3.46 | 0.05 | 0.17 |
| OTU97_24 (Prevotella) prevalence | RA \|\| id:ukb-b-11874 | ML | 2 | 50.22 | 25.33 | 0.05 | 0.97 |
| OTU99_1 (Bacteroides) prevalence | RA \|\| id:ukb-b-11874 | IVW(F) | 2 | 42.39 | 21.50 | 0.05 | 0.97 |
| OTU99_1 (Bacteroides) prevalence | RA \|\| id:ukb-b-11874 | IVW | 2 | 42.39 | 21.50 | 0.05 | 0.99 |
| OTU97_17 (Parabacteroides) abundance | RA \|\| id:ukb-b-11874 | IVW(F) | 2 | -17.19 | 8.72 | 0.05 | 0.97 |
| OTU97_17 (Parabacteroides) abundance | RA \|\| id:ukb-b-11874 | ML | 2 | -17.35 | 8.80 | 0.05 | 0.97 |
| OTU97_1 (Bacteroides) prevalence | RA \|\| id:ukb-b-11874 | IVW(F) | 2 | 42.32 | 21.52 | 0.05 | 0.97 |
| OTU97_1 (Bacteroides) prevalence | RA \|\| id:ukb-b-11874 | IVW | 2 | 42.32 | 21.52 | 0.05 | 0.99 |
| OTU99_1 (Bacteroides) prevalence | RA \|\| id:ukb-b-11874 | ML | 2 | 42.44 | 21.62 | 0.05 | 0.97 |

RA, rheumatoid arthritis; IVW, Inverse variance weighted; IVW(M), Inverse variance weighted (multiplicative random effects); IVW (F), Inverse variance weighted (F); ML, Maximum likelihood; nsnp is the number of SNPs being used as IVs, 95%CI: confidence interval, pval of the intercept from the method, p.adjust: the p value after using the Benjamini-Hochberg method; significant p.adjust were marked in bold.

**Table S4.** Mendelian randomization of metabolites and immune factors on rheumatoid arthritis in European population.

| **outcome** | **exposure** | **method** | **nsnp** | **b** | **se** | **pval** | **95%CI** | **p.adjust** |
| --- | --- | --- | --- | --- | --- | --- | --- | --- |
| **RA \|\| id:ukb-b-11874** | **X-11793--oxidized bilirubin*** | **IVW(M)** | **2** | **-0.002** | **3.59E-05** | **0.00E+00** | **0.998 (0.998, 0.998)** | **0.00E+00** |
| **RA \|\| id:ukb-b-11874** | **Alanine** | **IVW(M)** | **2** | **0.013** | **0.001** | **3.98E-149** | **1.013 (1.012, 1.014)** | **2.47E-147** |
| **RA \|\| id:ukb-b-11874** | **ADSGEGDFXAEGGGVR*** | **IVW(M)** | **2** | **0.001** | **2.45E-05** | **5.41E-131** | **1.001 (1.001, 1.001)** | **2.24E-129** |
| **RA \|\| id:ukb-b-11874** | **Uridine** | **IVW(M)** | **2** | **-0.022** | **0.003** | **4.45E-14** | **0.979 (0.973, 0.984)** | **1.38E-12** |
| **RA \|\| id:ukb-b-11874** | **4-acetamidobutanoate** | **IVW(M)** | **2** | **0.011** | **0.002** | **1.05E-11** | **1.011 (1.008, 1.014)** | **2.60E-10** |
| **RA \|\| id:ukb-b-11874** | **3-methyl-2-oxovalerate** | **IVW(M)** | **3** | **0.013** | **0.002** | **8.81E-10** | **1.013 (1.009, 1.017)** | **1.82E-08** |
| **RA \|\| id:ukb-b-11874** | **1,5-anhydroglucitol (1,5-AG)** | **IVW(M)** | **2** | **0.005** | **0.001** | **5.79E-05** | **1.005 (1.002, 1.007)** | **0.00102548** |
| RA \|\| id:ukb-b-11874 | Tyrosine | IVW(M) | 2 | 0.011 | 0.004 | 0.006 | 1.011 (1.003, 1.019) | 0.094 |
| RA \|\| id:ukb-b-11874 | Serine | IVW(M) | 2 | 0.001 | 2.98E-04 | 0.008 | 1.001 (1, 1.001) | 0.111 |
| RA \|\| id:ukb-b-11874 | Gamma-glutamylvaline | Wald ratio | 1 | -0.026 | 0.010 | 0.009 | 0.974 (0.956, 0.994) | 0.111 |
| RA \|\| id:ukb-b-11874 | 4-acetamidobutanoate | IVW(F) | 2 | 0.011 | 0.004 | 0.014 | 1.011 (1.002, 1.019) | 0.346 |
| RA \|\| id:ukb-b-11874 | 4-acetamidobutanoate | IVW | 2 | 0.011 | 0.004 | 0.014 | 1.011 (1.002, 1.019) | 0.375 |
| RA \|\| id:ukb-b-11874 | 4-acetamidobutanoate | Maximum likelihood | 2 | 0.011 | 0.004 | 0.015 | 1.011 (1.002, 1.019) | 0.466 |
| RA \|\| id:ukb-b-11874 | Gamma-glutamylphenylalanine | IVW(M) | 2 | 0.007 | 0.003 | 0.017 | 1.007 (1.001, 1.013) | 0.189 |
| RA \|\| id:ukb-b-11874 | X-11593--O-methylascorbate* | IVW(M) | 5 | -0.002 | 0.001 | 0.019 | 0.998 (0.997, 1) | 0.193 |
| RA \|\| id:ukb-b-11874 | Uridine | IVW(F) | 2 | -0.022 | 0.009 | 0.019 | 0.979 (0.961, 0.996) | 0.346 |
| RA \|\| id:ukb-b-11874 | Uridine | IVW | 2 | -0.022 | 0.009 | 0.019 | 0.979 (0.961, 0.996) | 0.375 |
| RA \|\| id:ukb-b-11874 | Laurate (12:0) | IVW(M) | 2 | 0.010 | 0.005 | 0.021 | 1.01 (1.002, 1.019) | 0.202 |
| RA \|\| id:ukb-b-11874 | 1-eicosatrienoylglycerophosphocholine* | Wald ratio | 1 | 0.014 | 0.006 | 0.024 | 1.014 (1.002, 1.027) | 0.202 |
| RA \|\| id:ukb-b-11874 | Uridine | Maximum likelihood | 2 | -0.022 | 0.010 | 0.025 | 0.979 (0.96, 0.997) | 0.466 |
| RA \|\| id:ukb-b-11874 | 3-methyl-2-oxovalerate | IVW(F) | 3 | 0.013 | 0.006 | 0.026 | 1.013 (1.002, 1.025) | 0.346 |
| RA \|\| id:ukb-b-11874 | 3-methyl-2-oxovalerate | IVW | 3 | 0.013 | 0.006 | 0.026 | 1.013 (1.002, 1.025) | 0.375 |
| RA \|\| id:ukb-b-11874 | X-14208--phenylalanylserine | Wald ratio | 1 | -0.004 | 0.002 | 0.026 | 0.996 (0.992, 0.999) | 0.202 |
| RA \|\| id:ukb-b-11874 | Octadecanedioate | Wald ratio | 1 | -0.012 | 0.005 | 0.027 | 0.989 (0.978, 0.999) | 0.202 |
| RA \|\| id:ukb-b-11874 | Acetylphosphate | Wald ratio | 1 | 0.031 | 0.014 | 0.028 | 1.031 (1.003, 1.06) | 0.202 |
| RA \|\| id:ukb-b-11874 | 3-methyl-2-oxovalerate | Maximum likelihood | 3 | 0.013 | 0.006 | 0.029 | 1.013 (1.001, 1.025) | 0.466 |
| RA \|\| id:ukb-b-11874 | 1-arachidonoylglycerophosphoethanolamine* | IVW(F) | 2 | 0.007 | 0.003 | 0.036 | 1.007 (1, 1.013) | 0.346 |
| RA \|\| id:ukb-b-11874 | Aspartylphenylalanine | Wald ratio | 1 | -0.006 | 0.003 | 0.036 | 0.995 (0.989, 1) | 0.214 |
| RA \|\| id:ukb-b-11874 | X-14189--leucylalanine | Wald ratio | 1 | -0.004 | 0.002 | 0.036 | 0.996 (0.992, 1) | 0.214 |
| RA \|\| id:ukb-b-11874 | X-14205--alpha-glutamyltyrosine | Wald ratio | 1 | -0.006 | 0.003 | 0.036 | 0.994 (0.989, 1) | 0.214 |
| RA \|\| id:ukb-b-11874 | X-14304--leucylalanine | Wald ratio | 1 | -0.005 | 0.002 | 0.036 | 0.995 (0.991, 1) | 0.214 |
| RA \|\| id:ukb-b-11874 | 1-arachidonoylglycerophosphoethanolamine* | Maximum likelihood | 2 | 0.007 | 0.003 | 0.037 | 1.007 (1, 1.013) | 0.466 |
| RA \|\| id:ukb-b-11874 | 3-methyl-2-oxovalerate | Weighted median | 3 | 0.014 | 0.007 | 0.044 | 1.014 (1, 1.027) | 0.772 |
| RA \|\| id:ukb-b-11874 | N-acetylornithine | Wald ratio | 1 | 0.002 | 0.001 | 0.049 | 1.002 (1, 1.003) | 0.276 |
| RA \|\| id:ukb-b-11874 | Complement C1q tumor necrosis factor-related protein 1 | IVW(M) | 2 | -1.45E-04 | 4.07E-06 | 4.58E-277 | 1 (1, 1) | 1.1E-275 |
| RA \|\| id:ukb-b-11874 | Tumor necrosis factor-inducible gene 6 protein | IVW(M) | 2 | -3.97E-04 | 1.23E-05 | 1.87E-229 | 1 (1, 1) | 2.24E-228 |

RA, rheumatoid arthritis; IVW, Inverse variance weighted; IVW(M), Inverse variance weighted (multiplicative random effects); IVW (F), Inverse variance weighted (fixed effects); nsnp is the number of SNPs being used as IVs, 95%CI: confidence interval, pval of the intercept from the method, p.adjust: the p value after using the Benjamini-Hochberg method; significant p.adjust were marked in bold.

**Table S5.** Mendelian randomization of rheumatoid arthritis on immune factors in European population.

| **outcome** | **exposure** | **method** | **nsnp** | **b** | **se** | **pval** | **p.adjust** |
| --- | --- | --- | --- | --- | --- | --- | --- |
| **Complement C1q tumor necrosis factor-related protein 3** | **RA \|\| id:ukb-b-11874** | **IVW(M)** | **2** | **-18.72** | **0.18** | **0** | **0** |
| **Tumor necrosis factor receptor superfamily member 14** | **RA \|\| id:ukb-b-11874** | **IVW(M)** | **2** | **-19.06** | **1.72** | **1.20E-28** | **4.24E-27** |
| **Interleukin-6 receptor subunit beta** | **RA \|\| id:ukb-b-11874** | **IVW(M)** | **2** | **-15.26** | **1.91** | **1.58E-15** | **3.75E-14** |
| **Complement C1q tumor necrosis factor-related protein 5** | **RA \|\| id:ukb-b-11874** | **IVW(M)** | **2** | **-7.86** | **1.18** | **2.86E-11** | **5.08E-10** |
| **Tumor necrosis factor receptor superfamily member 6B** | **RA \|\| id:ukb-b-11874** | **IVW(M)** | **2** | **5.83** | **0.88** | **3.62E-11** | **5.13E-10** |
| **Tumor necrosis factor ligand superfamily member 18** | **RA \|\| id:ukb-b-11874** | **IVW(M)** | **2** | **-28.20** | **4.39** | **1.29E-10** | **1.53E-09** |
| **Complement C1q tumor necrosis factor-related protein 1** | **RA \|\| id:ukb-b-11874** | **IVW(M)** | **2** | **-15.00** | **2.48** | **1.39E-09** | **1.41E-08** |
| Tumor necrosis factor ligand superfamily member 18 | RA \|\| id:ukb-b-11874 | IVW(F) | 2 | -28.20 | 9.85 | 0.004 | 0.26 |
| Tumor necrosis factor ligand superfamily member 18 | RA \|\| id:ukb-b-11874 | IVW | 2 | -28.20 | 9.85 | 0.004 | 0.30 |
| **Interleukin-6 receptor subunit alpha** | **RA \|\| id:ukb-b-11874** | **IVW(M)** | **2** | **21.28** | **7.49** | **0.005** | **0.04** |
| Tumor necrosis factor ligand superfamily member 18 | RA \|\| id:ukb-b-11874 | Maximum likelihood | 2 | -28.21 | 9.95 | 0.005 | 0.26 |
| **Tumor necrosis factor receptor superfamily member 27** | **RA \|\| id:ukb-b-11874** | **IVW(M)** | **2** | **17.79** | **6.39** | **0.005** | **0.04** |
| **Tumor necrosis factor receptor superfamily member 6** | **RA \|\| id:ukb-b-11874** | **IVW(M)** | **2** | **-21.21** | **7.79** | **0.006** | **0.05** |
| Interleukin-6 receptor subunit alpha | RA \|\| id:ukb-b-11874 | IVW(M) | 2 | 19.30 | 8.14 | 0.018 | 0.11 |
| Tumor necrosis factor receptor superfamily member 8 | RA \|\| id:ukb-b-11874 | IVW(F) | 2 | -22.95 | 9.88 | 0.020 | 0.26 |
| Tumor necrosis factor receptor superfamily member 8 | RA \|\| id:ukb-b-11874 | Maximum likelihood | 2 | -23.13 | 9.98 | 0.020 | 0.33 |
| Tumor necrosis factor ligand superfamily member 11 levels | RA \|\| id:ukb-b-11874 | Wald ratio | 1 | -79.98 | 35.16 | 0.023 | 0.14 |
| Tumor necrosis factor alpha levels | RA \|\| id:ukb-b-11874 | Wald ratio | 1 | 24.92 | 11.41 | 0.029 | 0.16 |
| Tumor necrosis factor ligand superfamily member 8 | RA \|\| id:ukb-b-11874 | IVW(F) | 2 | -21.41 | 9.85 | 0.030 | 0.26 |
| Tumor necrosis factor ligand superfamily member 8 | RA \|\| id:ukb-b-11874 | Maximum likelihood | 2 | -21.49 | 9.92 | 0.030 | 0.33 |
| Interleukin-6 receptor subunit alpha | RA \|\| id:ukb-b-11874 | IVW(F) | 2 | 21.28 | 9.85 | 0.031 | 0.26 |
| Interleukin-6 receptor subunit alpha | RA \|\| id:ukb-b-11874 | IVW | 2 | 21.28 | 9.85 | 0.031 | 0.32 |
| Interleukin-6 receptor subunit alpha | RA \|\| id:ukb-b-11874 | Maximum likelihood | 2 | 21.31 | 9.91 | 0.032 | 0.33 |
| Tumor necrosis factor receptor superfamily member 6 | RA \|\| id:ukb-b-11874 | IVW(F) | 2 | -21.21 | 9.88 | 0.032 | 0.26 |
| Tumor necrosis factor receptor superfamily member 6 | RA \|\| id:ukb-b-11874 | IVW | 2 | -21.21 | 9.88 | 0.032 | 0.32 |
| Tumor necrosis factor receptor superfamily member 6 | RA \|\| id:ukb-b-11874 | Maximum likelihood | 2 | -21.24 | 9.94 | 0.033 | 0.33 |
| Tumor necrosis factor beta levels | RA \|\| id:ukb-b-11874 | Wald ratio | 1 | -74.46 | 35.16 | 0.034 | 0.17 |
| Tumor necrosis factor receptor superfamily member 19L | RA \|\| id:ukb-b-11874 | IVW(F) | 2 | -20.10 | 9.85 | 0.041 | 0.26 |
| Tumor necrosis factor receptor superfamily member 19L | RA \|\| id:ukb-b-11874 | Maximum likelihood | 2 | -20.15 | 9.91 | 0.042 | 0.33 |
| Tumor necrosis factor receptor superfamily member 1A | RA \|\| id:ukb-b-11874 | IVW(M) | 2 | 2.46 | 1.21 | 0.043 | 0.19 |
| Tumor necrosis factor receptor superfamily member 11B | RA \|\| id:ukb-b-11874 | Maximum likelihood | 2 | -20.16 | 10.07 | 0.045 | 0.33 |
| Tumor necrosis factor receptor superfamily member 21 | RA \|\| id:ukb-b-11874 | IVW(M) | 2 | -18.37 | 9.19 | 0.046 | 0.19 |
| IL-20 | RA \|\| id:ukb-b-11874 | Wald ratio | 1 | -45.21 | 22.75 | 0.047 | 0.19 |
| Tumor necrosis factor receptor superfamily member 11B | RA \|\| id:ukb-b-11874 | IVW(F) | 2 | -19.56 | 9.88 | 0.048 | 0.26 |

RA, rheumatoid arthritis; IVW, Inverse variance weighted; IVW(M), Inverse variance weighted (multiplicative random effects); IVW (F), Inverse variance weighted (fixed effects); nsnp is the number of SNPs being used as IVs, 95%CI: confidence interval, pval of the intercept from the method, p.adjust: the p value after using the Benjamini-Hochberg method; significant p.adjust were marked in bold.

**Table S6.** The heterogeneity results from the Cochran's Q test and pleiotropy results from Egger intercept/MR-PROSSO analyses.

| **exposure** | **outcome** | **nsnp** | **population** | **Q** | **P-value** | **Egger intercept P-value** | **MR-PROSSO P-value** | **other** |
| --- | --- | --- | --- | --- | --- | --- | --- | --- |
| F_Bacteroidaceae abundance | RA | 7 | European | 2.460 | 0.873 | 0.300 | 0.851 |  |
| G_Alloprevotella prevalence | RA | 9 | European | 5.970 | 0.650 | 0.478 | 0.691 |  |
| G_Bacteroides abundance | RA | 7 | European | 2.456 | 0.873 | 0.300 | 0.851 |  |
| G_Bacteroidetes prevalence | RA | 2 | European | 0.023 | 0.878 |  |  |  |
| G_Prevotella abundance | RA | 6 | European | 1.459 | 0.918 | 0.840 | 0.926 |  |
| OTU97_34 (Ruminococcus) abundance | RA | 3 | European | 0.575 | 0.750 | 0.929 |  |  |
| OTU97_69 (Clostridiales) prevalence | RA | 4 | European | 1.047 | 0.790 | 0.997 | 0.815 |  |
| OTU99_110 (Clostridiales) abundance | RA | 6 | European | 5.446 | 0.364 | **0.047** | 0.496 | PMR-Egger>0.05 |
| OTU99_110 (Clostridiales) prevalence | RA | 2 | European | 0.100 | 0.751 |  |  |  |
| OTU99_5 (Sutterella) abundance | RA | 2 | European | 0.015 | 0.901 |  |  |  |
| OTU99_6 (Sutterella) prevalence | RA | 2 | European | 0.200 | 0.654 |  |  |  |
| OTU99_78 (Clostridiales) prevalence | RA | 4 | European | 1.051 | 0.789 | 0.993 | 0.815 |  |
| TestASV_32 (Ruminococcaceae) prevalence | RA | 3 | European | 0.211 | 0.890 | 0.732 |  |  |
| TestASV_4 (Alistipes) prevalence | RA | 3 | European | 0.232 | 0.890 | 0.954 |  |  |
| TestASV_6 (Subdoligranulum) abundance | RA | 2 | European | 0.024 | 0.877 |  |  |  |
| Uridine | RA | 2 | European | 0.096 | 0.756 |  |  |  |
| 1,5-anhydroglucitol (1,5-AG) | RA | 2 | European | 0.756 | 0.706 |  |  |  |
| 3-methyl-2-oxovalerate | RA | 3 | European | 0.264 | 0.876 | 0.976 |  |  |
| 4-acetamidobutanoate | RA | 2 | European | 0.132 | 0.717 |  |  |  |
| ADSGEGDFXAEGGGVR* | RA | 2 | European | 0.000 | 0.984 |  |  |  |
| Alanine | RA | 2 | European | 0.005 | 0.946 |  |  |  |
| X-11793--oxidized bilirubin* | RA | 2 | European | 0.001 | 0.978 |  |  |  |
| Complement C1q tumor necrosis factor-related protein 1 | RA | 1 | European | 0.000 | 0.992 |  |  |  |
| Tumor necrosis factor-inducible gene 6 protein | RA | 2 | European | 0.003 | 0.954 |  |  |  |

RA, rheumatoid arthritis; nsnp is the number of SNPs being used as IVs; significant p was marked in bold.

**Table S7.** Steiger test results from exposure to outcome.

| **exposure** | **outcome** | **r2_exp** | **r2_out** | **correct_causal_direction** | **steiger_test** | **population** |
| --- | --- | --- | --- | --- | --- | --- |
| F_Bacteroidaceae abundance | RA\|\| id:ukb-b-11874 | 0.016 | 1.405E-05 | TRUE | 1.03E-31 | European |
| G_Bacteroides abundance | RA\|\| id:ukb-b-11874 | 0.016 | 1.405E-05 | TRUE | 1.03E-31 | European |
| G_Prevotella abundance | RA\|\| id:ukb-b-11874 | 0.014 | 1.732E-05 | TRUE | 1.44E-27 | European |
| OTU97_34 (Ruminococcus) abundance | RA\|\| id:ukb-b-11874 | 0.007 | 1.292E-05 | TRUE | 3.68E-14 | European |
| OTU99_110 (Clostridiales) abundance | RA\|\| id:ukb-b-11874 | 0.014 | 3.527E-05 | TRUE | 1.05E-26 | European |
| OTU99_5 (Sutterella) abundance | RA\|\| id:ukb-b-11874 | 0.005 | 3.312E-06 | TRUE | 6.47E-11 | European |
| TestASV_6 (Subdoligranulum) abundance | RA\|\| id:ukb-b-11874 | 0.005 | 2.824E-06 | TRUE | 8.26E-11 | European |
| G_Alloprevotella prevalence | RA\|\| id:ukb-b-11874 | 0.021 | 3.726E-05 | TRUE | 4.96E-39 | European |
| G_Bacteroidetes prevalence | RA\|\| id:ukb-b-11874 | 0.005 | 2.044E-06 | TRUE | 3.45E-10 | European |
| OTU97_69 (Clostridiales) prevalence | RA\|\| id:ukb-b-11874 | 0.010 | 1.164E-05 | TRUE | 6.32E-19 | European |
| OTU99_110 (Clostridiales) prevalence | RA\|\| id:ukb-b-11874 | 0.005 | 6.892E-06 | TRUE | 9.09E-10 | European |
| OTU99_6 (Sutterella) prevalence | RA\|\| id:ukb-b-11874 | 0.005 | 1.353E-05 | TRUE | 1.50E-09 | European |
| OTU99_78 (Clostridiales) prevalence | RA\|\| id:ukb-b-11874 | 0.010 | 1.164E-05 | TRUE | 6.15E-19 | European |
| TestASV_32 (Ruminococcaceae) prevalence | RA\|\| id:ukb-b-11874 | 0.007 | 3.072E-06 | TRUE | 1.86E-14 | European |
| TestASV_4 (Alistipes) prevalence | RA\|\| id:ukb-b-11874 | 0.007 | 4.988E-06 | TRUE | 1.19E-14 | European |
| X-11793--oxidized bilirubin* | RA\|\| id:ukb-b-11874 | 0.075 | 4.181E-06 | TRUE | 1.82E-108 | European |
| Alanine | RA\|\| id:ukb-b-11874 | 0.012 | 6.695E-06 | TRUE | 6.44E-20 | European |
| ADSGEGDFXAEGGGVR* | RA\|\| id:ukb-b-11874 | 0.028 | 5.365E-07 | TRUE | 1.87E-24 | European |
| Uridine | RA\|\| id:ukb-b-11874 | 0.008 | 1.211E-05 | TRUE | 4.93E-14 | European |
| 4-acetamidobutanoate | RA\|\| id:ukb-b-11874 | 0.043 | 1.354E-05 | TRUE | 1.98E-62 | European |
| 3-methyl-2-oxovalerate | RA\|\| id:ukb-b-11874 | 0.019 | 1.132E-05 | TRUE | 6.61E-29 | European |
| 1,5-anhydroglucitol (1,5-AG) | RA\|\| id:ukb-b-11874 | 0.031 | 5.360E-06 | TRUE | 8.14E-51 | European |
| Complement C1q tumor necrosis factor-related protein 1 | RA\|\| id:ukb-b-11874 | 0.039 | 2.650E-07 | TRUE | 2.60E-30 | European |
| Tumor necrosis factor-inducible gene 6 protein | RA\|\| id:ukb-b-11874 | 0.162 | 7.473E-06 | TRUE | 3.08E-130 | European |

RA, rheumatoid arthritis.

**Table S8.** Colocalization analysis where loci provide evidence of a shared causal variant.

| **exposure** | **nsnp** | **PP.H0.abf** | **PP.H1.abf** | **PP.H2.abf** | **PP.H3.abf** | **PP.H4.abf** | **population** |
| --- | --- | --- | --- | --- | --- | --- | --- |
| OTU99_5 (Sutterella) abundance | 2 | 0.302 | 2.13E-06 | 0.696 | 2.48E-06 | 2.43E-03 | European |
| TestASV_6 (Subdoligranulum) abundance | 2 | 0.046 | 2.31E-06 | 0.651 | 1.94E-06 | 2.39E-03 | European |
| G_Bacteroidetes prevalence | 2 | 0.583 | 3.40E-06 | 0.416 | 1.25E-06 | 1.18E-03 | European |
| OTU99_110 (Clostridiales) prevalence | 2 | 0.608 | 6.30E-06 | 0.390 | 2.22E-06 | 1.83E-03 | European |
| OTU99_6 (Sutterella) prevalence | 2 | 0.608 | 6.30E-06 | 0.390 | 2.22E-06 | 1.83E-03 | European |
| G_Prevotella abundance | 6 | 0.274 | 8.88E-06 | 0.722 | 1.93E-05 | 4.12E-03 | European |
| OTU97_34 (Ruminococcus) abundance | 3 | 0.274 | 8.88E-06 | 0.722 | 1.93E-05 | 4.12E-03 | European |
| TestASV_4 (Alistipes) prevalence | 3 | 0.425 | 4.81E-06 | 0.573 | 4.16E-06 | 2.31E-03 | European |
| G_Alloprevotella prevalence | 9 | 0.229 | 2.16E-05 | 0.761 | 6.21E-05 | 9.62E-03 | European |
| OTU99_78 (Clostridiales) prevalence | 4 | 0.339 | 7.81E-06 | 0.656 | 1.07E-05 | 4.37E-03 | European |
| OTU97_69 (Clostridiales) prevalence | 4 | 0.339 | 7.81E-06 | 0.656 | 1.07E-05 | 4.37E-03 | European |
| TestASV_32 (Ruminococcaceae) prevalence | 3 | 0.489 | 4.55E-06 | 0.509 | 3.26E-06 | 1.48E-03 | European |
| G_Bacteroides abundance | 7 | 0.161 | 5.59E-06 | 0.836 | 2.65E-05 | 2.58E-03 | European |
| OTU99_110 (Clostridiales) abundance | 6 | 0.161 | 5.59E-06 | 0.836 | 2.65E-05 | 2.58E-03 | European |
| F_Bacteroidaceae abundance | 7 | 0.161 | 5.59E-06 | 0.837 | 2.65E-05 | 2.58E-03 | European |
| X-11793--oxidized bilirubin* | 2 | 0.161 | 5.59E-06 | 0.837 | 2.65E-05 | 2.58E-03 | European |
| Alanine | 2 | 0.000 | 6.65E-13 | 0.994 | 4.05E-06 | 5.74E-03 | European |
| ADSGEGDFXAEGGGVR* | 2 | 0.000 | 9.74E-13 | 0.997 | 2.47E-06 | 2.67E-03 | European |
| Uridine | 2 | 0.009 | 1.85E-07 | 0.982 | 1.06E-05 | 8.94E-03 | European |
| 4-acetamidobutanoate | 2 | 0.009 | 1.85E-07 | 0.982 | 1.06E-05 | 8.94E-03 | European |
| 3-methyl-2-oxovalerate | 3 | 0.000 | 3.05E-15 | 0.991 | 8.73E-06 | 8.65E-03 | European |
| 1,5-anhydroglucitol (1,5-AG) | 2 | 0.000 | 3.05E-15 | 0.991 | 8.73E-06 | 8.65E-03 | European |
| Complement C1q tumor necrosis factor-related protein 1 | 1 | 0.000 | 1.32E-17 | 0.997 | 2.33E-06 | 2.53E-03 | European |
| Tumor necrosis factor-inducible gene 6 protein | 2 | 0.000 | 2.25E-113 | 0.989 | 2.85E-06 | 1.12E-02 | European |

RA, rheumatoid arthritis; nsnp is the number of SNPs being used as IVs; PP.H4.abf > 0·8 were marked in bold.

**Table S9.** Mendelian randomization of metabolites and immune factors on rheumatoid arthritis in European population.

| **outcome** | **exposure** | **method** | **nsnp** | **b** | **se** | **pval** | **95%CI** | **p.adjust** |
| --- | --- | --- | --- | --- | --- | --- | --- | --- |
| **Alanine** | **OTU99_5 (Sutterella) abundance** | **IVW(M)** | **3** | **-0.011** | **0.001** | **6.17E-82** | **0.989 (0.988, 0.99)** | **2.22E-79** |
| **3-methyl-2-oxovalerate** | **TestASV_4 (Alistipes) prevalence** | **IVW(M)** | **4** | **0.013** | **0.003** | **5.03E-07** | **1.013 (1.008, 1.018)** | **2.65E-05** |
| **3-methyl-2-oxovalerate** | **OTU99_78 (Clostridiales) prevalence** | **IVW(M)** | **2** | **-0.022** | **0.005** | **2.03E-05** | **0.978 (0.969, 0.988)** | **7.70E-04** |
| **3-methyl-2-oxovalerate** | **OTU97_69 (Clostridiales) prevalence** | **IVW(M)** | **2** | **-0.022** | **0.005** | **4.21E-05** | **0.978 (0.968, 0.989)** | **0.001** |
| **X-11793--oxidized bilirubin*** | **TestASV_32 (Ruminococcaceae) prevalence** | **IVW(M)** | **6** | **-0.010** | **0.003** | **1.00E-04** | **0.99 (0.985, 0.995)** | **0.003** |
| **ADSGEGDFXAEGGGVR*** | **TestASV_32 (Ruminococcaceae) prevalence** | **IVW(M)** | **6** | **0.016** | **0.005** | **3.91E-04** | **1.016 (1.007, 1.025)** | **0.010** |
| ADSGEGDFXAEGGGVR* | TestASV_32 (Ruminococcaceae) prevalence | IVW(F) | 6 | 0.016 | 0.006 | 0.005 | 1.016 (1.005, 1.028) | 0.797 |
| ADSGEGDFXAEGGGVR* | TestASV_32 (Ruminococcaceae) prevalence | IVW | 6 | 0.016 | 0.006 | 0.005 | 1.016 (1.005, 1.028) | 1.000 |
| 4-acetamidobutanoate | G_Alloprevotella prevalence | IVW(F) | 5 | -0.012 | 0.004 | 0.006 | 0.988 (0.979, 0.997) | 0.808 |
| 4-acetamidobutanoate | G_Alloprevotella prevalence | Maximum likelihood | 5 | -0.013 | 0.005 | 0.006 | 0.987 (0.978, 0.996) | 0.954 |
| ADSGEGDFXAEGGGVR* | TestASV_32 (Ruminococcaceae) prevalence | Maximum likelihood | 6 | 0.017 | 0.006 | 0.006 | 1.017 (1.005, 1.029) | 0.954 |
| ADSGEGDFXAEGGGVR* | OTU99_110 (Clostridiales) prevalence | IVW(M) | 4 | 0.025 | 0.009 | 0.007 | 1.025 (1.007, 1.043) | 0.095 |
| 3-methyl-2-oxovalerate | OTU99_78 (Clostridiales) prevalence | IVW(F) | 2 | -0.022 | 0.009 | 0.011 | 0.978 (0.962, 0.995) | 0.843 |
| 3-methyl-2-oxovalerate | OTU99_78 (Clostridiales) prevalence | IVW | 2 | -0.022 | 0.009 | 0.011 | 0.978 (0.962, 0.995) | 1.000 |
| 3-methyl-2-oxovalerate | OTU97_69 (Clostridiales) prevalence | IVW(F) | 2 | -0.022 | 0.009 | 0.011 | 0.978 (0.962, 0.995) | 0.843 |
| 3-methyl-2-oxovalerate | OTU97_69 (Clostridiales) prevalence | IVW | 2 | -0.022 | 0.009 | 0.011 | 0.978 (0.962, 0.995) | 1.000 |
| Alanine | G_Bacteroidetes prevalence | IVW(M) | 3 | 0.002 | 0.001 | 0.014 | 1.002 (1, 1.003) | 0.159 |
| 3-methyl-2-oxovalerate | OTU99_78 (Clostridiales) prevalence | Maximum likelihood | 2 | -0.022 | 0.009 | 0.017 | 0.978 (0.961, 0.996) | 0.954 |
| 3-methyl-2-oxovalerate | OTU97_69 (Clostridiales) prevalence | Maximum likelihood | 2 | -0.022 | 0.009 | 0.018 | 0.978 (0.961, 0.996) | 0.954 |
| Alanine | TestASV_4 (Alistipes) prevalence | IVW(M) | 4 | 0.007 | 0.003 | 0.026 | 1.007 (1.001, 1.014) | 0.243 |
| ADSGEGDFXAEGGGVR* | TestASV_32 (Ruminococcaceae) prevalence | Simple median | 6 | 0.017 | 0.008 | 0.027 | 1.017 (1.002, 1.032) | 1.000 |
| 4-acetamidobutanoate | OTU99_5 (Sutterella) abundance | IVW(M) | 3 | 0.009 | 0.004 | 0.028 | 1.009 (1.001, 1.017) | 0.258 |
| Alanine | OTU99_6 (Sutterella) prevalence | Maximum likelihood | 4 | 0.012 | 0.006 | 0.040 | 1.013 (1.001, 1.025) | 0.954 |
| 4-acetamidobutanoate | G_Alloprevotella prevalence | IVW(M) | 5 | -0.012 | 0.006 | 0.041 | 0.988 (0.976, 0.999) | 0.323 |
| 4-acetamidobutanoate | G_Alloprevotella prevalence | IVW | 5 | -0.012 | 0.006 | 0.041 | 0.988 (0.976, 0.999) | 1.000 |
| Alanine | OTU99_6 (Sutterella) prevalence | IVW(F) | 4 | 0.011 | 0.006 | 0.046 | 1.012 (1, 1.023) | 0.923 |
| X-11793--oxidized bilirubin* | TestASV_32 (Ruminococcaceae) prevalence | IVW(F) | 6 | -0.010 | 0.005 | 0.047 | 0.99 (0.981, 1) | 0.926 |
| X-11793--oxidized bilirubin* | TestASV_32 (Ruminococcaceae) prevalence | IVW | 6 | -0.010 | 0.005 | 0.047 | 0.99 (0.981, 1) | 1.000 |
| X-11793--oxidized bilirubin* | TestASV_32 (Ruminococcaceae) prevalence | Maximum likelihood | 6 | -0.010 | 0.005 | 0.050 | 0.99 (0.98, 1) | 0.954 |
| **Tumor necrosis factor-inducible gene 6 protein** | **G_Bacteroidetes prevalence** | **IVW(M)** | **7** | **0.114** | **0.025** | **0.000** | **1.12 (1.066, 1.177)** | **0.002** |
| **Tumor necrosis factor-inducible gene 6 protein** | **TestASV_6 (Subdoligranulum) abundance** | **IVW(M)** | **8** | **0.143** | **0.039** | **0.000** | **1.153 (1.069, 1.244)** | **0.028** |
| Complement C1q tumor necrosis factor-related protein 1 | OTU99_6 (Sutterella) prevalence | IVW(M) | 10 | 0.098 | 0.037 | 0.007 | 1.103 (1.027, 1.185) | 0.251 |
| Tumor necrosis factor-inducible gene 6 protein | OTU99_5 (Sutterella) abundance | IVW(M) | 8 | 0.060 | 0.023 | 0.009 | 1.062 (1.015, 1.112) | 0.279 |
| Complement C1q tumor necrosis factor-related protein 1 | OTU99_6 (Sutterella) prevalence | Maximum likelihood | 10 | 0.102 | 0.043 | 0.016 | 1.108 (1.019, 1.204) | 0.937 |
| Complement C1q tumor necrosis factor-related protein 1 | OTU99_6 (Sutterella) prevalence | IVW(F) | 10 | 0.098 | 0.041 | 0.018 | 1.103 (1.017, 1.196) | 0.904 |
| Complement C1q tumor necrosis factor-related protein 1 | OTU99_6 (Sutterella) prevalence | IVW | 10 | 0.098 | 0.041 | 0.018 | 1.103 (1.017, 1.196) | 1.000 |
| Complement C1q tumor necrosis factor-related protein 1 | OTU99_6 (Sutterella) prevalence | Simple median | 10 | 0.124 | 0.054 | 0.021 | 1.132 (1.019, 1.257) | 1.000 |
| Complement C1q tumor necrosis factor-related protein 1 | OTU99_6 (Sutterella) prevalence | Weighted median | 10 | 0.120 | 0.054 | 0.027 | 1.128 (1.014, 1.254) | 1.000 |
| Complement C1q tumor necrosis factor-related protein 1 | G_Bacteroidetes prevalence | IVW(M) | 7 | -0.068 | 0.032 | 0.032 | 0.935 (0.879, 0.994) | 0.487 |
| Tumor necrosis factor-inducible gene 6 protein | TestASV_6 (Subdoligranulum) abundance | IVW(F) | 8 | 0.143 | 0.067 | 0.035 | 1.153 (1.01, 1.316) | 0.922 |
| Tumor necrosis factor-inducible gene 6 protein | TestASV_6 (Subdoligranulum) abundance | IVW | 8 | 0.143 | 0.067 | 0.035 | 1.153 (1.01, 1.316) | 1.000 |
| Tumor necrosis factor-inducible gene 6 protein | TestASV_6 (Subdoligranulum) abundance | Maximum likelihood | 8 | 0.144 | 0.069 | 0.036 | 1.155 (1.01, 1.322) | 0.937 |
| Tumor necrosis factor-inducible gene 6 protein | G_Bacteroidetes prevalence | IVW(F) | 7 | 0.114 | 0.055 | 0.037 | 1.12 (1.007, 1.247) | 0.922 |
| Tumor necrosis factor-inducible gene 6 protein | G_Bacteroidetes prevalence | IVW | 7 | 0.114 | 0.055 | 0.037 | 1.12 (1.007, 1.247) | 1.000 |
| Tumor necrosis factor-inducible gene 6 protein | G_Bacteroidetes prevalence | Maximum likelihood | 7 | 0.114 | 0.056 | 0.039 | 1.121 (1.006, 1.25) | 0.937 |
| Tumor necrosis factor-inducible gene 6 protein | TestASV_6 (Subdoligranulum) abundance | Simple median | 8 | 0.171 | 0.084 | 0.042 | 1.186 (1.006, 1.398) | 1.000 |

RA, rheumatoid arthritis; IVW, Inverse variance weighted; IVW(M), Inverse variance weighted (multiplicative random effects); IVW (F), Inverse variance weighted (fixed effects); nsnp is the number of SNPs being used as IVs, 95%CI: confidence interval, pval of the intercept from the method, p.adjust: the p value after using the Benjamini-Hochberg method; significant p.adjust were marked in bold.

**Table S10.** Two-step Mendelian randomization analysis of rheumatoid arthritis in European population.

| **Pathway** | **exposure→outcome** | | | **exposure→mediation** | | | **mediation→RA** | | | **Indirect effect: exposure→mediation→RA** | | | | | |
| --- | --- | --- | --- | --- | --- | --- | --- | --- | --- | --- | --- | --- | --- | --- | --- |
|  | **θ0** | | | **θ1** | | | **θ2** | | | **θ3** | | | |  |  |
|  | **beta** | **se** | **p** | **beta** | **se** | **p** | **beta** | **se** | **p** | **beta** | **se** | **p** | **Proportion mediated (%)** | | **Directionally consistent** |
| OTU99_5 (Sutterella) abundance → Alanine → RA | -0.0007 | 6.93E-05 | 2.74E-23 | -0.011 | 0.001 | 6.17E-82 | 1.30E-02 | 5.01E-04 | 3.98E-149 | -1.45E-04 | 9.42E-06 | 9.81E-54 | | 21.10 | Y |
| TestASV_4 (Alistipes) prevalence → 3-methyl-2-oxovalerate → RA | 0.0007 | 1.59E-04 | 2.19E-05 | 0.013 | 0.003 | 5.03E-07 | 1.31E-02 | 2.13E-03 | 8.81E-10 | 1.67E-04 | 4.30E-05 | 1.02E-04 | | 24.73 | Y |
| OTU99_78 (Clostridiales) prevalence → 3-methyl-2-oxovalerate → RA | -0.0005 | 1.40E-04 | 4.27E-04 | -0.022 | 0.005 | 2.03E-05 | 1.31E-02 | 2.13E-03 | 8.81E-10 | -2.86E-04 | 8.18E-05 | 4.67E-04 | | 58.22 | **Y** |
| OTU97_69 (Clostridiales) prevalence → 3-methyl-2-oxovalerate → RA | -0.0005 | 1.39E-04 | 4.12E-04 | -0.022 | 0.005 | 4.21E-05 | 1.31E-02 | 2.13E-03 | 8.81E-10 | -2.86E-04 | 8.40E-05 | 6.61E-04 | | 58.07 | **Y** |
| TestASV_32 (Ruminococcaceae) prevalence → ADSGEGDFXAEGGGVR* → RA | -0.0002 | 7.21E-05 | 6.71E-04 | 0.016 | 0.005 | 3.91E-04 | 5.96E-04 | 2.45E-05 | 5.41E-131 | 9.66E-06 | 2.75E-06 | 4.49E-04 | | -3.94 | N |
| TestASV_32 (Ruminococcaceae) prevalence → X-11793--oxidized bilirubin* → RA | -0.0002 | 7.21E-05 | 6.71E-04 | -0.010 | 0.003 | 1.00E-04 | -1.82E-03 | 3.59E-05 | 0.00E+00 | 1.81E-05 | 4.66E-06 | 1.05E-04 | | -7.38 | N |
| TestASV_6 (Subdoligranulum) abundance → Tumor necrosis factor-inducible gene 6 protein → RA | -0.0007 | 9.91E-05 | 1.76E-13 | 0.143 | 0.039 | 2.43E-04 | -3.97E-04 | 1.23E-05 | 1.87E-229 | -5.65E-05 | 1.55E-05 | 2.67E-04 | | 7.75 | Y |
| G_Bacteroidetes prevalence → Tumor necrosis factor-inducible gene 6 protein → RA | 0.0005 | 7.40E-05 | 5.18E-10 | 0.114 | 0.025 | 7.44E-06 | -3.97E-04 | 1.23E-05 | 1.87E-229 | -4.50E-05 | 1.01E-05 | 9.07E-06 | | -9.79 | N |

RA, rheumatoid arthritis; both mediation analysis methods showed that the direction of the mediation is consistent with the direction of the exposure-RA were marked in bold.

**Table S11.** Multivariable Mendelian randomization analysis of rheumatoid arthritis in European population.

| **Pathway** | **Direct effect from MVMR** | | | | | | **Total effect from univariable MR** | | | **Indirect effect from MVMR** | | | | |
| --- | --- | --- | --- | --- | --- | --- | --- | --- | --- | --- | --- | --- | --- | --- |
|  | **beta** | **se** | **p** | **Q** | **Q_pval** | **F** | **beta** | **se** | **p** | **beta** | **se** | **p** | **Proportion mediated (%)** | **Directionally consistent** |
| OTU99_5 (Sutterella) abundance → Alanine → RA | -7.66E-04 | 6.33E-04 | 0.23 | 2.48 | 0.29 | 14.74 | -6.89E-04 | 6.93E-05 | 2.74E-23 | 7.73E-05 | 6.37E-04 | 0.10 | -11.22 | N |
| TestASV_4 (Alistipes) prevalence → 3-methyl-2-oxovalerate → RA | 6.81E-04 | 3.97E-04 | 0.09 | 2.87 | 0.58 | 26.88 | 6.76E-04 | 1.59E-04 | 2.19E-05 | -4.87E-06 | 4.28E-04 | 0.01 | -0.72 | N |
| OTU99_78 (Clostridiales) prevalence → 3-methyl-2-oxovalerate → RA | -2.52E-04 | 4.14E-04 | 0.54 | 3.38 | 0.34 | 9.07 | -4.92E-04 | 1.40E-04 | 4.27E-04 | -2.40E-04 | 4.37E-04 | 0.42 | 48.70 | **Y** |
| OTU97_69 (Clostridiales) prevalence → 3-methyl-2-oxovalerate → RA | -2.32E-04 | 4.12E-04 | 0.57 | 3.44 | 0.33 | 28.87 | -4.93E-04 | 1.39E-04 | 4.12E-04 | -2.61E-04 | 4.35E-04 | 0.45 | 52.89 | **Y** |
| TestASV_32 (Ruminococcaceae) prevalence → ADSGEGDFXAEGGGVR* → RA | -1.90E-04 | 5.86E-05 | 0.00 | 0.10 | 0.98 | 14.42 | -2.45E-04 | 7.21E-05 | 6.71E-04 | -5.50E-05 | 9.29E-05 | 0.45 | 22.44 | Y |
| TestASV_32 (Ruminococcaceae) prevalence → X-11793--oxidized bilirubin* → RA | -1.58E-04 | 4.79E-05 | 0.00 | 0.07 | 0.97 | 15.18 | -2.45E-04 | 7.21E-05 | 6.71E-04 | -8.68E-05 | 8.65E-05 | 0.68 | 35.40 | Y |
| TestASV_6 (Subdoligranulum) abundance → Tumor necrosis factor-inducible gene 6 protein → RA | -8.53E-04 | 3.81E-04 | 0.03 | 0.67 | 0.72 | 84.96 | -7.30E-04 | 9.91E-05 | 1.76E-13 | 1.23E-04 | 3.94E-04 | 0.25 | -16.91 | N |
| G_Bacteroidetes prevalence → Tumor necrosis factor-inducible gene 6 protein → RA | 4.36E-04 | 3.50E-04 | 0.21 | 1.03 | 0.60 | 13.97 | 4.60E-04 | 7.40E-05 | 5.18E-10 | 2.37E-05 | 3.58E-04 | 0.05 | 5.16 | Y |

RA, rheumatoid arthritis; both mediation analysis methods showed that the direction of the mediation is consistent with the direction of the exposure-RA were marked in bold.

**Table S12.** Mendelian randomization of microbiota on rheumatoid arthritis (*P* < 5×10^-8^).

| **outcome** | **exposure** | **method** | **nsnp** | **b** | **se** | **pval** | **95%CI** | **p.adjust** | **population** |
| --- | --- | --- | --- | --- | --- | --- | --- | --- | --- |
| RA \|\| id:ukb-b-11874 | TestASV_27 (Ruminococcaceae) abundance | Wald ratio | 1 | -1.13E-03 | 5.65E-04 | 0.05 | 0.999 (0.998, 1) | 0.43 | European |
| RA \|\| id:ukb-b-11874 | C_Clostridia abundance | Wald ratio | 1 | 1.42E-03 | 1.44E-03 | 0.32 | 1.001 (0.999, 1.004) | 0.96 | European |
| RA \|\| id:ukb-b-11874 | OTU99_40 (Proteobacteria) abundance | Wald ratio | 1 | -2.84E-04 | 1.12E-03 | 0.80 | 1 (0.998, 1.002) | 0.96 | European |
| RA \|\| id:ukb-b-11874 | OTU99_558 (Bacteroidales) abundance | Wald ratio | 1 | 4.00E-04 | 7.49E-04 | 0.59 | 1 (0.999, 1.002) | 0.96 | European |
| RA \|\| id:ukb-b-11874 | OTU99_92 (Ruminococcus) prevalence | Wald ratio | 1 | -2.29E-04 | 6.06E-04 | 0.71 | 1 (0.999, 1.001) | 0.96 | European |
| RA \|\| id:ukb-b-11874 | OTU99_32 (Prevotella) abundance | Wald ratio | 1 | 1.53E-04 | 7.95E-04 | 0.85 | 1 (0.999, 1.002) | 0.96 | European |
| RA \|\| id:ukb-b-11874 | OTU97_257 (Mitsuokella) prevalence | Wald ratio | 1 | 1.15E-18 | 4.74E-19 | 0.02 | 1 (1, 1) | 0.30 | European |
| RA \|\| id:ukb-b-11874 | TestASV_22 (Sutterella) abundance | Wald ratio | 1 | 1.34E-04 | 7.98E-04 | 0.87 | 1 (0.999, 1.002) | 0.96 | European |
| RA \|\| id:ukb-b-11874 | TestASV_16 (Bacteroides) prevalence | Wald ratio | 1 | -3.83E-04 | 4.27E-04 | 0.37 | 1 (0.999, 1) | 0.96 | European |
| RA \|\| id:ukb-b-11874 | OTU97_80 (Ruminococcus) prevalence | Wald ratio | 1 | -2.01E-04 | 6.12E-04 | 0.74 | 1 (0.999, 1.001) | 0.96 | European |
| RA \|\| id:ukb-b-11874 | OTU97_34 (Ruminococcus) prevalence | Wald ratio | 1 | 3.06E-05 | 5.64E-04 | 0.96 | 1 (0.999, 1.001) | 0.96 | European |
| RA \|\| id:ukb-b-11874 | TestASV_26 (Phascolarctobacterium) abundance | Wald ratio | 1 | 5.64E-04 | 3.17E-04 | 0.07 | 1.001 (1, 1.001) | 0.47 | European |
| RA \|\| id:ukb-b-11874 | OTU99_94 (Bacteroides) prevalence | Wald ratio | 1 | 1.14E-04 | 3.01E-04 | 0.70 | 1 (1, 1.001) | 0.96 | European |
| RA \|\| id:ukb-b-11874 | OTU97_95 (Cytophagales) prevalence | Wald ratio | 1 | 2.31E-20 | 1.15E-19 | 0.84 | 1 (1, 1) | 0.96 | European |
| RA \|\| id:ukb-b-11874 | G_Alloprevotella abundance | Wald ratio | 1 | -6.85E-04 | 6.21E-04 | 0.27 | 0.999 (0.998, 1.001) | 0.96 | European |
| RA \|\| id:ukb-b-11874 | OTU99_108 (Cytophagales) prevalence | Wald ratio | 1 | 2.31E-20 | 1.15E-19 | 0.84 | 1 (1, 1) | 0.96 | European |
| RA \|\| id:ukb-b-11874 | OTU99_35 (Ruminococcus) prevalence | Wald ratio | 1 | 3.05E-05 | 5.63E-04 | 0.96 | 1 (0.999, 1.001) | 0.96 | European |
| RA \|\| id:ukb-b-11874 | TestASV_4 (Alistipes) abundance | Wald ratio | 1 | -2.73E-04 | 1.08E-03 | 0.80 | 1 (0.998, 1.002) | 0.96 | European |
| RA \|\| id:ukb-b-11874 | OTU97_82 (Bacteroides) prevalence | Wald ratio | 1 | 1.15E-04 | 3.03E-04 | 0.70 | 1 (1, 1.001) | 0.96 | European |

RA, rheumatoid arthritis; nsnp is the number of SNPs being used as IVs, 95%CI: confidence interval, pval of the intercept from the method, p.adjust: the p value after using the Benjamini-Hochberg method; significant p.adjust were marked in bold.
